# Demand heterogeneity in insurance markets: Implications for equity and efficiency

**DOI:** 10.3982/qe794

**Published:** 2017-11-20

**Authors:** MICHAEL GERUSO

**Affiliations:** Department of Economics, University of Texas at Austin and NBER

**Keywords:** Community rating, adverse selection, demand heterogeneity

## Abstract

In many markets insurers are barred from price discrimination based on consumer characteristics like age, gender, and medical history. In this paper, I build on a recent literature to show why such policies are inefficient if consumers differ in their willingness-to-pay for insurance conditional on the insured losses they generate. Using administrative claims data, I then show that this type of demand heterogeneity is empirically relevant in a consumer health plan setting. Younger and older consumers and men and women reveal strikingly different demand for health insurance, conditional on their objective medical spending risk. This implies that these groups must face different prices so as to sort themselves efficiently across insurance contracts. The theoretical and empirical analysis highlights a fundamental trade-off between equity and efficiency that is unique to selection markets.

## 1. INTRODUCTION

In many insurance markets in the United States and abroad, insurers face restrictions against setting premiums based on observable consumer characteristics like age, sex, and claims history. Such restrictions have been in place for employer-sponsored health plans in the United States since 1974. In recent years, these types of nondiscrimination rules have become more widely adopted across insurance market settings. For example, plans in the Affordable Care Act (ACA) exchanges cannot price-discriminate based on sex or medical history, and there are binding restrictions on how age can enter pricing, with premium differences capped at a ratio of 3 : 1 across age groups in most states. In the same spirit, the European Union’s high court ruled in 2011 that sex cannot enter premium determination in health insurance, life insurance, or annuities, even though sex is a strong predictor of insurer costs in these markets.

It is well known that these types of regulations aimed at promoting equity can exacerbate asymmetric information problems. This is because even though insurers can easily observe consumer characteristics that predict costs, they are constrained by regulators to act as if these characteristics were unobservable in setting premiums. This leaves markets susceptible to full or partial unravelling, as more costly consumers select into more generous contracts (Akerlof (1970)).^1^ For this reason, nondiscrimination policies are generally accompanied by complementary regulations like consumer tax subsidies and insurer subsidies in the form of risk adjustment. In the ACA exchanges, Medicare Advantage, Medicare Part D, and insurance markets around the world, these policy tools are used to counteract selection distortions that uniform pricing could otherwise introduce. The conventional wisdom is that with the right subsidies, uniform pricing carries no efficiency cost relative to any other feasible pricing policy.^2^ This view is supported by much of the early literature in risk adjustment, including Cutler and Reber (1998), Van de Ven and Ellis (2000), and Glazer and McGuire (2000).

In this paper, I build on recent work by Glazer and McGuire (2011) and Bundorf, Levin, and Mahoney (2012) that shows why this conventional wisdom is wrong, and I develop some previously unexplored implications of demand heterogeneity in the context of nondiscrimination policies. In selection markets, if consumers differ in their valuations of insurance contracts conditional on the costs they generate for insurers, then discriminatory pricing can represent a feasible welfare improvement over the best nondiscriminatory pricing. The intuition is straightforward: Efficient prices are determined by the intersection of the willingness-to-pay and marginal cost curves. Therefore, if groups of buyers like men and women, rich and poor, or young and old have systematically different willingness-to-pay for insurance holding expected losses (i.e., insurer marginal costs) fixed, then the prices that would induce efficient self-sorting must be different across these groups. This is a unique feature of selection markets. Unlike other consumer goods, the producer’s marginal cost of generating an insurance contract is fundamentally tied to the characteristics of the particular consumer who purchases the contract. The consumers generate both the demand and the cost curves.

Under what conditions would this kind of demand heterogeneity exist? First, consumers who represent the same actuarial risk to insurers may simply differ in their attitudes toward risk and therefore willingness-to-pay for insurance (Finkelstein and McGarry (2006), Cohen and Einav (2007), Fang, Keane, and Silverman (2008)). Second, consumers with identical risk preferences who face the same expected losses may nonetheless differ in the spread of their risk distributions, a possibility often assumed away in simple foundational models like Rothschild and Stiglitz (1976) that consider a binary insurable loss. Or, even assuming identical risk preferences and identical insurable risk profiles, if utility does not take the constant absolute risk aversion (CARA) form, differences in wealth will drive differences in willingness-to-pay for insurance.^3^ The model of this paper nests these and many other potential drivers of demand heterogeneity that are ignored under the conventional notion of efficiency in insurance markets because that notion implicitly assumes a one-to-one mapping from insurable risk to insurance valuation.

In the paper, I begin by adapting the canonical model of insurance choice to accommodate optimal insurance pricing under heterogenous demand. The main theoretical result is that when demand differs across observable consumer groups, so too must prices so as to achieve the best feasible sorting of consumers into contracts. The intuition behind the result is illustrated in a series of simple figures, extending the now-standard graphical selection frameworks in Cutler and Reber (1998), Feldman and Dowd (2000), Einav and Finkelstein (2011), and Hackmann, Kolstad, and Kowalski (2015). I then develop a simple sufficient statistics test to detect the relevant kind of demand heterogeneity. This test has minimal data and identification requirements, and can be implemented broadly. The econometrician or regulator need observe only equilibrium plan choices and claims data.

I demonstrate the empirical relevance of the model using detailed administrative health claims data from a large employer that allow precise controls for expected and realized healthcare spending. These data show that there is substantial demand heterogeneity across easily identifiable demographic groups. Willingness-to-pay for the more generous insurance option is strongly correlated with age and sex, even after conditioning on medical spending risk. For instance, 50- to 59-year-old workers in this setting are 50% more likely than 18- to 29-year-old workers to choose more insurance, holding expected medical spending fixed at any level. The theoretical framework of the paper makes it clear that these facts alone—without additional identifying assumptions—are sufficient to indicate that implementing age-specific pricing would yield a welfare improvement. The demand patterns, which are revealed in simple semiparametric plots of plan choices versus administrative claims costs, stand in stark contrast to conventional wisdom, which would imply that young and old would differ in demand only because they differed in insurable risk and which would predict that take-up conditional on insurable risk would be identical across the two groups.

Despite significant theoretical and empirical research attention to selection in recent years, the implications of heterogeneous demand in selection markets have not been fully explored. Only a small prior literature has recognized that first-best allocations cannot be achieved under asymmetric information with demand heterogeneity. Einav and Finkelstein (2011) note the phenomenon, and Glazer and McGuire (2011) and Bundorf, Levin, and Mahoney (2012) address it in more depth, with the latter establishing the general result that a first-best is infeasible if demand heterogeneity exists. With the sole exception of a stylized treatment in Glazer and McGuire (2011), past studies have only considered market segmentation according to consumer cost types.^4^ The key innovation of this paper is to show that although a first-best is infeasible, there is a feasible welfare improvement over nondiscriminatory pricing that can be achieved by segmenting the market according to consumer *preference* types. Importantly, I show that the result holds even in the extreme (but conceptually simple) case of segmenting two groups that have different preferences but identical marginal cost curves.

To complement the sufficient statistics approach that constitutes the main empirical analysis, I also adapt a standard (e.g., Handel (2013), Handel, Kolstad, and Spinnewijn (2015)) expected utility model of insurance choice. The additional structure allows me to (i) show that the data match the model in terms of across- and within-group demand heterogeneity, (ii) show that constrained optimal prices vary across demographic groups, and (iii) estimate the misallocation of consumers across plans due to nondiscrimination policies. I find that older consumers and women optimally face higher premiums, and as a result younger consumers and men are suboptimally underinsured when facing (constrained) optimal uniform prices. In the specific choice setting I examine, optimal prices for the oldest consumers are about 2.4 times those for the youngest consumers. The unique insight of this paper is that optimal pricing differs not because these groups face different expected health spending, which they do. With standard tools like consumer subsidies, uniform prices could sort all consumers perfectly, regardless of expected costs. Rather, optimal prices differ because, on average, consumers in these groups value insurance contracts differently when they face the same risk.

The general theoretical phenomenon I describe is likely to be broadly relevant. Empirical work across a variety of insurance market settings has shown that consumer demand for insurance can deviate significantly from the underlying insurable risk. Cutler, Finkelstein, and McGarry (2008) provide a survey of this literature and suggest that “heterogeneity in preferences may be as, or more, important than heterogeneity in risk in explaining insurance demand.” The prior research has almost exclusively focused on preference heterogeneity that is correlated with risk (positively or negatively). The present paper advances this literature in a new direction, showing why the part of demand heterogeneity that is *uncorrelated* with risk also has important implications in these markets. This is the key departure from prior work.

The central finding of this paper—that a social planner setting prices in insurance markets would want to set different prices for different groups of consumers—also contrasts sharply with current policy in the United States and abroad, which has trended toward nondiscriminatory prices. While regulators and policymakers may nonetheless wish to pursue equity objectives, this paper makes clear that there is an unavoidable efficiency cost of doing so.^5^ Subsequent work that builds on the insight of the present paper, including Layton, Ellis, and McGuire (2015), has begun to take this explicitly into account. The findings here also highlight the loss inherent in using insurance price regulation as a redistributive mechanism (Finkelstein, Poterba, and Rothschild (2009)).

The rest of the paper proceeds as follows. Section 2 models the relevant phenomenon and then develops the novel insights regarding price discrimination. This section also outlines the sufficient statistic test. Section 3 describes the data, and Section 4 presents a set of reduced-form empirical results that demonstrate that willingness-to-pay for insurance varies systematically with age and sex for reasons unrelated to health spending. Section 5 estimates a structural model of plan choice that builds on the basic reduced-form findings. Finally, in Section 6, the structural parameter estimates are used to infer the welfare impacts of alternative pricing policies that allow for discrimination. Additional material is available in supplementary files on the journal website, http://qeconomics.org/supp/794/supplement.pdf and http://qeconomics.org/supp/794/code_and_data.zip.

## 2. DEMAND HETEROGENEITY IN INSURANCE MARKETS

In this section I highlight the efficiency problem created by demand heterogeneity, within the context of the standard model of insurance market selection. I then develop the result that a social planner setting prices (or a regulator setting subsidies) can improve welfare by setting prices that are a function of some observable characteristic that is correlated with revealed consumer preferences. This holds even if that characteristic is not correlated with the insurable risk. Finally, I develop a simple empirical test that uses commonly available claims data to reveal whether pricing-relevant demand heterogeneity exists in a market.

### 2.1 The canonical model

Consider consumers i∈I, who are described by characteristics partitioned into two vectors, δ and ψ. The vector δ describes any consumer characteristics that affect the insurer’s costs of providing insurance, including the consumer’s health risk. The vector ψ contains all other consumer characteristics relevant for insurance choice, such as wealth and risk preferences. Insurance contracts j∈J are described by prices p and plan features ϕ, where ϕ includes networks, copays, deductibles, and so forth.

Uncertain future health expenditures are made in various states of the world s∈S, where the probability distribution over health states for person i is $G_{i}=G\left(s|\delta_{i}\right)$. The expected utility of a contract $\left(p_{j},\phi_{j}\right)$ to a consumer $\left(\delta_{i},\psi_{i}\right)$ is

(1) $$v\left(p_{j},\phi_{j},\delta_{i},\psi_{i}\right)=\int u\left(s,p_{j},\phi_{j},\delta_{i},\psi_{i}\right)\cdot G\left(s|\delta_{i}\right)ds.$$

This setup is intended to closely track the “canonical model” of insurance choice in Einav, Finkelstein, and Levin (2010).^6^ With expected utility defined as above, consumers choose plans that generate the highest expected utility:

(2) $$v\left(p_{j},\phi_{j},\delta_{i},\psi_{i}\right)\geq v\left(p_{k},\phi_{k},\delta_{i},\psi_{i}\right)\;\forall k\in J.$$

Risk-neutral insurers incur costs due to claims paid to providers. Expected costs to the insurer, $c_{ij}$, depend on state-specific health events and plan characteristics like deductibles,

(3) $$c\left(\phi_{j},\delta_{i}\right)=\int \tau \left(s,\phi_{j},\delta_{i}\right)\cdot G\left(s|\delta_{i}\right)ds,$$

where τ expresses state-specific insurer costs. Note that the insurer’s expected costs depend on consumer characteristics that determine the distribution of health risk $G\left(s|\delta_{i}\right)$ but not on preferences or wealth, which are contained in $\psi_{i}$.

Define total social surplus as $W=\sum_{i\in I}\sum_{j\in J}I\left(j_{i}\right)\cdot \left(v\left(p_{j},\phi_{j},\delta_{i},\psi_{i}\right)-c\left(\phi_{j},\delta_{i}\right)\right)$, where $I\left(j_{i}\right)$ is an indicator function for person i being enrolled in plan j. Maximizing W requires that consumers sort to plans where their valuation is in the greatest excess of the cost of providing insurance. This is the standard (unconstrained) efficiency condition.^7^ It is typically invoked to illustrate how, in markets with adverse selection, competitive equilibria are inefficient without regulatory interventions, such as consumer or insurer subsidies.

### 2.2 Preference heterogeneity in the canonical model

Without departing from the canonical model, it is straightforward to observe that the surplus generated by a plan choice can differ between individuals i and $i^{\prime }$ who would generate the same expected cost to insure. In particular, the preference parameter ψ enters v but not c, so that $c\left(\phi_{j},\delta_{i}\right)=c\left(\phi_{j},\delta_{i^{\prime }}\right)$ does not imply $v\left(p_{j},\phi_{j},\delta_{i},\psi_{i}\right)=v\left(p_{j},\phi_{j},\delta_{i^{\prime }},\psi_{i^{\prime }}\right)$. In other words, there is no one-to-one mapping between the v and c functions.

It is not difficult to conceive of quantitatively important demand heterogeneity remaining after conditioning on expected costs. Higher risk aversion, captured in ψ, will correspond to higher willingness-to-pay for insurance, holding fixed the actual risk distribution that a consumer faces. There is indeed substantial empirical evidence of heterogeneity in preferences over insurance purchase (e.g., Finkelstein and McGarry (2006), Cohen and Einav (2007), Fang, Keane, and Silverman (2008), Cutler, Finkelstein, and McGarry (2008)). The conceptual framework also nests other drivers of variation in willingness-to-pay for insurance, holding $c_{ij}$ fixed. For instance, if risk preferences are identical across consumers but utility does not take the constant absolute risk aversion (CARA) form commonly assumed in empirical insurance studies, then differences in income will drive heterogeneity in v conditional on c. Similarly, if assets at risk vary, for example due to bankruptcy laws that act as implicit insurance (Mahoney (2015)), then so too will consumers’ valuation of an insurance contract, even when faced with identical preferences and identical health risk, G(s), and when such consumers would generate identical costs to the insurer.

Alternatively, even in the case when both preferences ψ and expected insurer losses $c_{ij}$ are identical between consumers, valuations can nonetheless differ if the risk distributions, G, differ in higher moments. If $\tilde{G}$ is a mean-preserving spread of G, then an otherwise similar consumer facing $\tilde{G}$ will value insurance more highly than a consumer facing G. This is true even though expected insurer (and social resource) costs are identical for the $\tilde{G}$ and G cases.

In general, the welfare-maximizing insurance allocation may differ across consumers among whom c is equal for a variety of plausible reasons. The sufficient statistic test that follows in Section 2.5 is intended to capture any such reason.

### 2.3 Implications for efficient sorting

**FIGURE 1.**
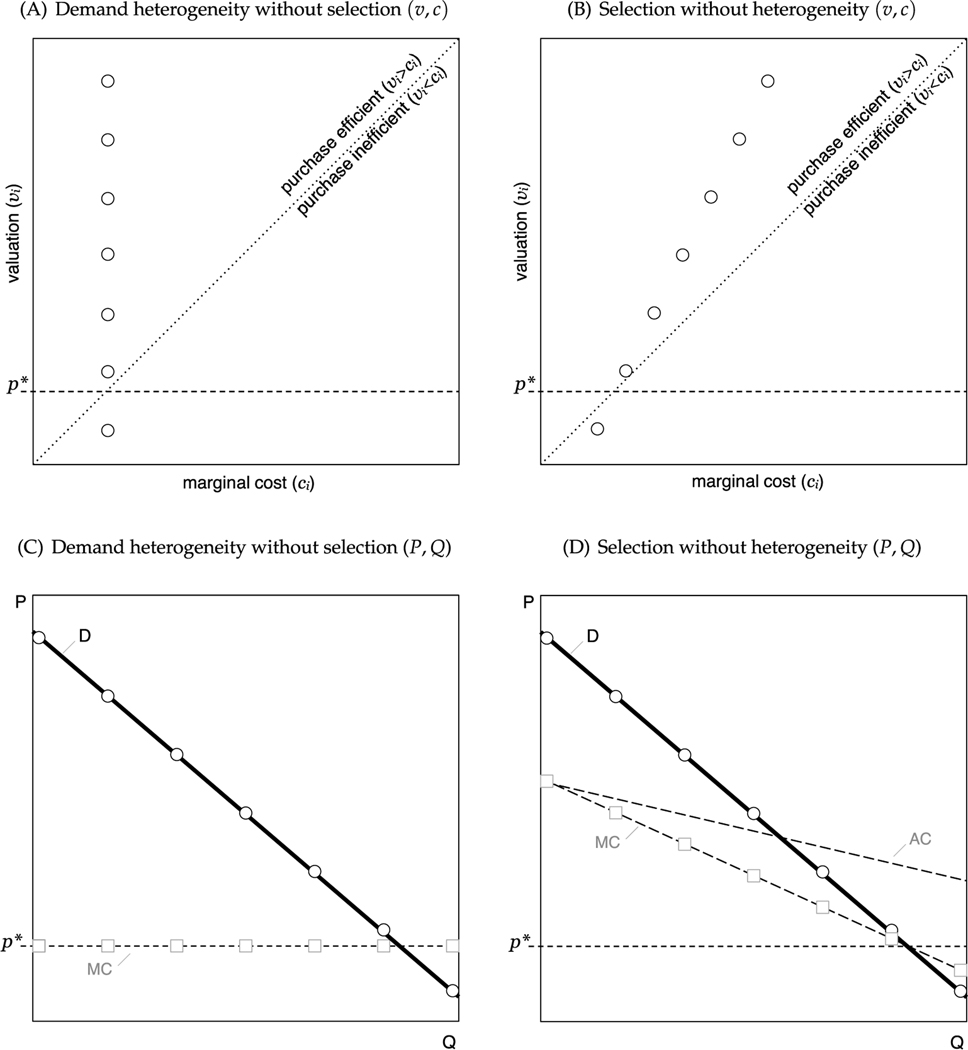
Selection or demand heterogeneity: A single price can sort efficiently. The figure establishes the baseline cases of demand heterogeneity without selection (panels A and C) and of selection without demand heterogeneity (panels B and D). Circles represent individuals, with the vertical axes in the top panels measuring plan valuation, v, and the horizontal axes measuring the insurer’s expected costs of covering claims, c. Selection implies correlation between c and v. Panels C and D plot the demand and cost curves implied by the cost and valuation pairs plotted in panels A and B. Valuations are plotted as circles and costs as squares in the bottom panels, and quantity along the horizontal axes is scaled from 0 to 100%. Demand heterogeneity is defined as consumer valuations that vary after conditioning on the insurer’s expected costs. In these plots, this would imply multiple vertical positions for some fixed horizontal position in (v,c) space, as in panel A. The 45-degree line separates the cases in which purchasing insurance is socially efficient from those in which it is efficient to remain uninsured. Consumers make efficient choices if and only if v≥c, and choose to take up insurance if and only if v≥p. In these baseline cases, a single price $p^{*}$ sorts all consumers efficiently.

**FIGURE 2.**
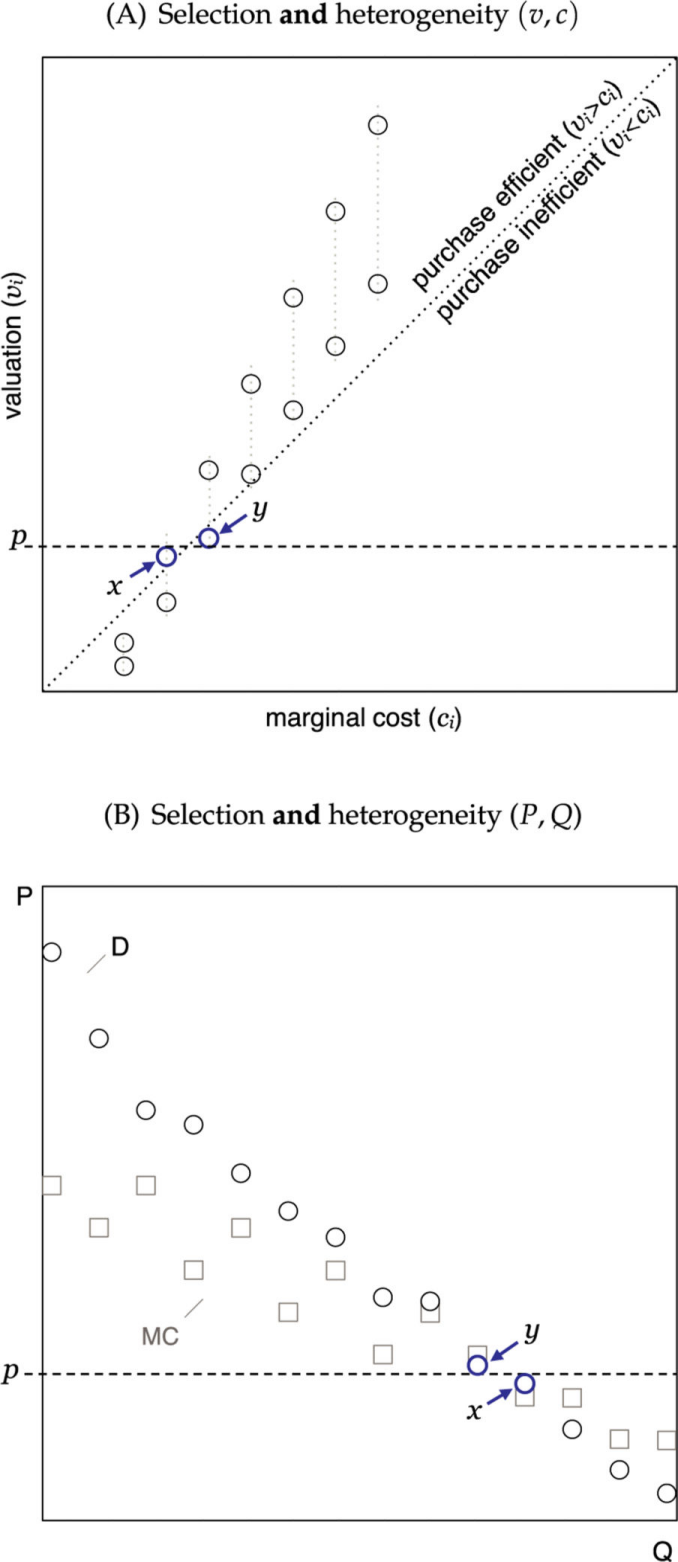
Selection and demand heterogeneity: No single price sorts efficiently. The figure shows how selection and demand heterogeneity interact in selection markets. The panels are constructed as in Figure 1, but now simultaneously incorporate demand heterogeneity and selection. Under these conditions, no single price can sort all consumers efficiently. Price would need to be higher than the depicted price p to sort consumer y efficiently, but lower than p to sort consumer x efficiently. In the corresponding demand diagram of panel B, demand declines monotonically, but the costs implied by panel A lead to a nonmonotonic marginal cost “cloud” because there is no longer a one-to-one mapping of consumer valuations to marginal costs. See the notes to Figure 1 for additional documentation.

Figures 1 and 2 illustrate the important interplay of selection and demand heterogeneity in a series of simple plots. Before illustrating in Figure 2 how selection and demand heterogeneity interact, I first reference in Figure 1 two familiar baseline cases: heterogeneity without selection in panel A, and selection without heterogeneity in panel B.

The top panels of Figure 1 plot joint distributions of $v\left(\phi_{j},\delta_{i},\psi_{i}\right)$ and $c\left(\phi_{j},\delta_{i}\right)$ associated with some insurance contract j. Valuations, abbreviated $v_{i}$, are along the vertical axes, and expected costs, $c_{i}$, are along the horizontal axes. The valuations and costs are relative to an outside option, which, for the purpose of considering optimal pricing, can be assumed to be no insurance.^8^ For simplicity, each point in these plots can be interpreted as representing the contract valuation and expected cost pair of an individual consumer.^9^ The heterogeneity of interest occurs when $v_{i}$ varies across individuals, holding $c_{i}$ fixed.

A consumer purchases the contract if and only if $v_{i}\geq p$. A price $p^{*}$ is efficient if it satisfies $v_{i}\geq p^{*}$ if and only if $v_{i}\geq c_{i}$. In the figure, a dashed 45-degree line separates the space into efficient contracting above the 45-degree line $\left(v_{i}\geq c_{i}\right)$ and efficient noncontracting below it $\left(v_{i}<c_{i}\right)$. Horizontal lines in Figure 1 correspond to prices that induce consumers to self-sort into efficient choices.

Panel A corresponds to a typical goods market. There is heterogenous willingness-to-pay, leading to downward sloping demand, but there is no inherent relationship between $v_{i}$ and $c_{i}$. Panel C plots the corresponding demand diagram. Note that the apparent uniformity of marginal costs in panel A of Figure 1 is not intended to rule out nonconstant marginal costs at the industry or firm level, but it does assume that the particular consumer purchasing the contract does not affect the producer’s marginal costs.^10^

Panel B of Figure 1 corresponds to the framework most widely applied to guide empirical work on selection. For ease of comparison, it is constructed to generate the same demand curve as in panel A. In contrast to panel A, there is no demand heterogeneity, but there is selection: Costs in panel B are systematically related to valuations. This represents the special feature of insurance markets with asymmetric information: The firm’s costs of producing the good are a function of the characteristics of consumers purchasing the goods and are therefore linked to consumers’ valuations. In particular, the joint distribution is constructed here such that higher valuations are associated with higher costs, generating adverse selection on price. In the corresponding demand diagram in panel D, this selection generates downward sloping demand and marginal cost curves. Panel D of Figure 1 mirrors the graphical frameworks in Einav, Finkelstein, and Cullen (2010), Hackmann, Kolstad, and Kowalski (2015), and other recent studies, which in turn echo Cutler and Reber (1998) and Feldman and Dowd (2000) and ultimately adapt the intuition of the Akerlof (1970) lemons model.

Much of the recent empirical literature on selection in insurance markets has built on the intuition embodied in panel D to demonstrate the extent to which perfect competition, which generates average cost pricing, induces inefficient consumer sorting or market unravelling, or in the case of Starc (2014), that imperfect competition can act as a countervailing force against this unravelling. One important takeaway from this “textbook” selection model is that while inefficient average cost pricing naturally occurs in competitive markets, proper interventions (such as consumer subsidies) can induce efficient sorting in competitive markets without sacrificing the equity goal of making premiums independent of health state, age, or gender.^11^

Because the intention is to show the conditions under which prices can induce efficient sorting of consumers, assume that a social planner observes the joint distributions over (v,c) and can arbitrarily set prices, but—consistent with the notion of asymmetric information—does not observe $c_{i}$ for any specific individual, and therefore cannot price as a function of unobservable consumer costs. It is nonetheless true that in each of the panels in Figure 1, it is possible to draw a single price that sorts all consumers efficiently. Horizontal lines at $p^{*}$ in the figure show the efficient price.

Now consider Figure 2, which introduces the feature of interest: selection interacting with demand heterogeneity. For each level of costs along the horizontal axis in panel A, there exist two different valuations. In this case there is no price that can induce efficient sorting. To see this, note the candidate price p plotted as a dashed horizontal line. Prices must be lower than p to sort type x efficiently. But it must be higher than p to sort type y efficiently. Clearly, no single price can satisfy these criteria simultaneously. The allocative efficiency problem can also be seen in the corresponding demand diagram in panel B of Figure 2. No horizontal price line can be drawn such that $v_{i}\geq p$ if and only if $v_{i}\geq c_{i}$.^12^

In the presence of demand heterogeneity, marginal costs no longer trace a single curve but rather become a cloud of points. Because there is no unique intersection point between this cloud and the demand curve, there is no uniform price that generates efficient consumption.^13^

The general point about the inefficiency of price as a sorting mechanism for selection markets with demand heterogeneity has been briefly noted by Einav and Finkelstein (2011) and explored in more detail by Bundorf, Levin, and Mahoney (2012) and Glazer and McGuire (2011). Panel A of Figure 2, in fact, closely parallels a plot in Bundorf, Levin, and Mahoney (2012). However, with the exception of Glazer and McGuire (2011), the literature has focused on pricing on some signal of costs or on finding the second-best response in terms of a uniform price, like p in Figure 2. In the next section, I show that there may be a feasible improvement relative to the best uniform price that involves pricing on a signal of preferences. Developing this finding theoretically and then demonstrating that such cases may be empirically relevant are the main goals of this paper.

### 2.4 Price discrimination on preferences

A feasible, welfare-improving refinement relative to the best uniform price may be possible if there exists an observable correlate of demand. “Feasible” here means that costs remain unobservable and cannot be used in pricing, consistent with the notion of asymmetric information. I begin with graphical intuition and then formalize the finding.

**FIGURE 3.**
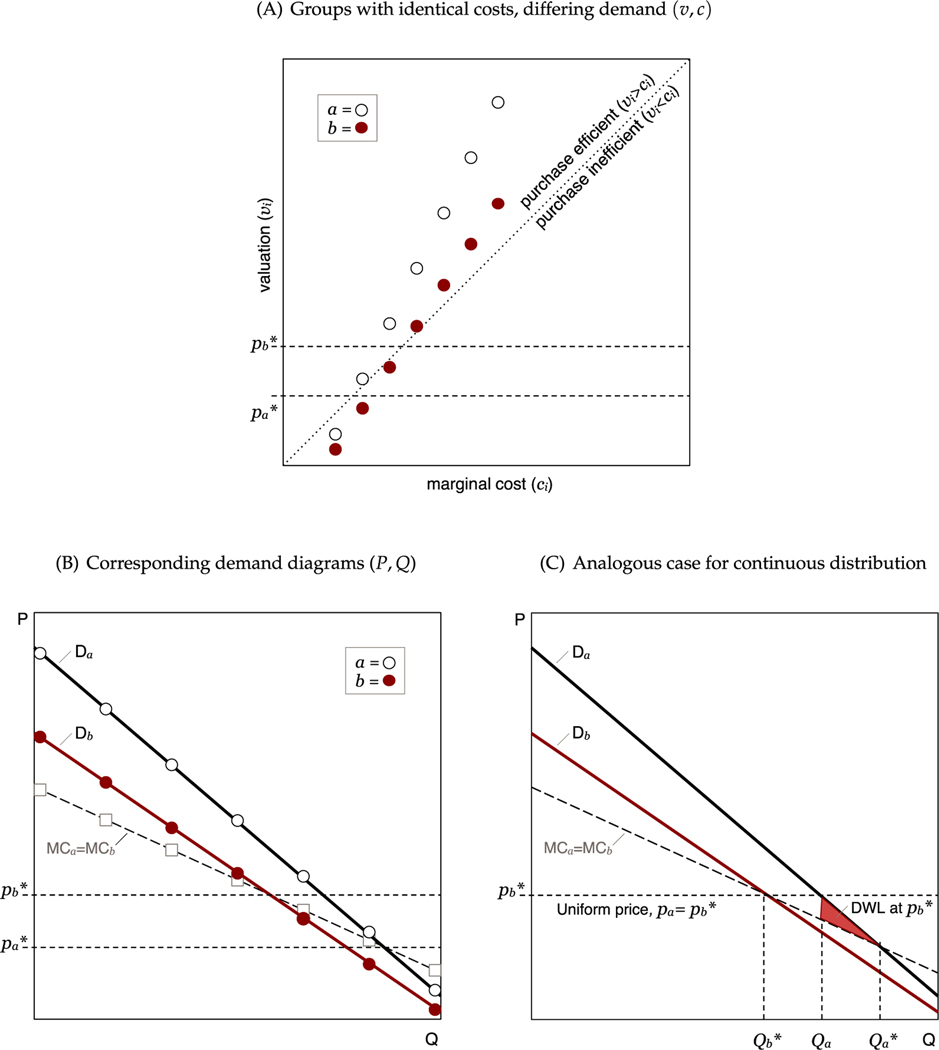
Price discrimination on preferences can be welfare-improving, even if costs are identical. This figure modifies Figure 2 to allow for identifiable consumer types, labeled a and b, while maintaining Figure 2’s exact distribution of (v,c) pairs overall. In the empirical application, identifiable types are defined by sex and age, which vary in both willingness-to-pay and costs. For simplicity, the graphical example here is constructed so that the types vary only in willingness–to-pay, v, and generate identical distributions of costs. Prices $p_{a}^{*}$ and $p_{b}^{*}$, respectively, sort types a and b efficiently. Thus price discrimination represents a feasible improvement over the best uniform price from Figure 2. Panel C plots the demand diagram for a case like panel B, but with a large set of consumers and a continuous distribution of costs within each type. In panel C, the shaded triangle depicts the welfare loss associated with setting a uniform price for all consumers at the level that is optimal for the b types, $p_{b}^{*}$. See the notes to Figures 1 and 2 for additional documentation.

Panel A of Figure 3 repeats panel A of Figure 2 with one difference: The scatter points representing individuals are now identifiable as belonging to two observable groups: group a is represented by hollow circles; group b is represented by solid circles. The identifiable types could represent income, wealth, sex, age, or any other observable. It is important to note, however, that by construction, the two groups exhibit identical risk distributions (i.e., the distribution of the circles along the horizontal axis). This construction isolates the phenomenon of interest, which is heterogeneity in preferences or demand, not heterogeneity in costs. The consumer groups a and b differ only in willingness-to-pay for insurance.

In the case of Figure 3, it is straightforward to see that a welfare-improving policy is to segment the market by preference type, setting different prices for groups a and b.^14^ The prices $p_{a}^{*}$ and $p_{b}^{*}$ that sort all consumers efficiently are depicted in the figure. In panel B, two separate demand diagrams for the a and b markets are overlaid. The overlay highlights that selection within the a and b markets produces exactly the same marginal cost curves, but differences in v conditional on c generate different demand and therefore different optimal pricing. Despite the fact that the joint distribution of valuations and costs are exactly the same as in panel A of Figure 2, the market segmentation allows prices to efficiently sort consumers. Intuitively, by segmenting the market according to consumer preference types, the nonmonotone cloud of points that comprise the marginal cost curve are separated into monotone cost curves within each new market. Panel C shows the result for an analogous case with a large number of consumers in each group and continuous distributions of costs.

To formalize the intuition of Figure 3, consider an observable characteristic z that is contained in consumer characteristics ψ and/or δ and is therefore correlated with willingness-to-pay. If z remains correlated with willingness-to-pay after conditioning on expected costs, then with some additional assumptions on single crossing and submarket monotonicity described in Appendix A.1, optimal pricing within market segments partitioned along the characteristic z is welfare-improving relative to the optimal uniform price. The proof in Appendix A.1 follows the same intuition as the diagram in Figure 3: Prices that induce efficient allocations are set where the demand and cost curves cross, and since these curves have different crossing points among groups for which v systematically differs conditional on c, segmenting the market by the characteristic z raises total surplus.

The novel idea of the model is that price discrimination across groups with systematically different preferences is optimal. This is not because these groups face different insurable risk (which by construction they do not here) but rather because these groups value insurance contracts differently conditional on facing the same risk.^15^ This is the key insight of the model that is distinct from other work that has considered segmenting selection markets according to *cost* types.

For completeness, Appendix Figure A1 graphs the case that corresponds most closely to the notion that young and old, rich and poor, men and women, and so forth, would differ in insurance demand only because of cost differences. It is straightforward to observe in Figure A1 that under this assumption, costs perfectly align with valuations, and therefore even if types could be identified and the market segmented, there would be no welfare gain to price discrimination, relative to the constrained optimal uniform price.

### 2.5 A sufficient statistic test

The last section discussed consumer valuations of insurance contracts, but the econometrician observes insurance choices, not underlying valuations. The natural analogue to the partial correlation between observables and plan valuations $\left(\rho_{v,z|c}\right)$ is the partial correlation between observables and plan choices $\left(\rho_{I(j),z|c}\right)$.

Let I(z=a) and I(z=b) represent indicator functions denoting membership in groups a and b, and let I(j) indicate enrollment in plan j. By revealed preference, satisfying the inequality with respect to plan choices

(4) E[I(j)∣c,I(z=a)]≠E[I(j)∣c,I(z=b)]

is a sufficient condition for identifying differences in plan valuations between types a and b.^16^ If the condition in (4) is met, then by the logic of Section 2.4 price discrimination on z yields a feasible improvement relative to the best uniform price.^17^ As I illustrate in the next section, checking the condition in expression (4) is simple with the kind of information readily available in health claims data.

Expression (4) is is not a test for selection. Although (4) parallels the widely applied Chiappori and Salanie (2000) test for selection in insurance markets in terms of form and simplicity, it answers a very different question. Abstracting from moral hazard, the Chiappori and Salanie test and its many implementations (e.g., Finkelstein and Poterba (2004)) is a test for positive correlation between purchasing a more generous contract and relevant loss-related outcomes, such as the claims costs borne by insurers. It takes a form like E[c∣I(j)]≠E[c∣I(k)] and asks whether there is selection on costs across plans. The test in (4) indicates nothing about selection on costs. Instead, it asks whether one group of consumers reveals different preferences over plans, holding the cost they generate for the insurer fixed.

Implementing the test in (4) requires generating an estimate, ${\hat{c}}_{i}$, of each individual’s expected costs, $c_{i}$. Because most observable consumer characteristics (e.g., age, sex, income, wealth) will be correlated both with contract valuations and also with expected costs, it is important to demonstrate empirically that the control ${\hat{c}}_{i}$ is sufficient to remove any partial correlation between $z_{i}$ and $c_{i}$. I discuss the practical issues of estimation in detail in Section 3.3 after introducing the data.

### 2.6 Incorporating more complex heterogeneity

The case in Figure 3 is a conceptually useful starting point, but two complications are likely to be relevant in real-world settings, including in the empirical setting of this paper. First, identifiable groups will likely differ from each other not only in valuations but also in risk profiles. Second, residual demand heterogeneity will always exist within whatever groups can be identified for segmentation. For example, as I show in Section 6, women have greater demand for insurance than men, holding expected losses fixed, but within the male and female groups there remains substantial idiosyncratic heterogeneity in revealed preference. It is essentially this within-group heterogeneity that Bundorf, Levin, and Mahoney (2012) study.

**FIGURE 4.**
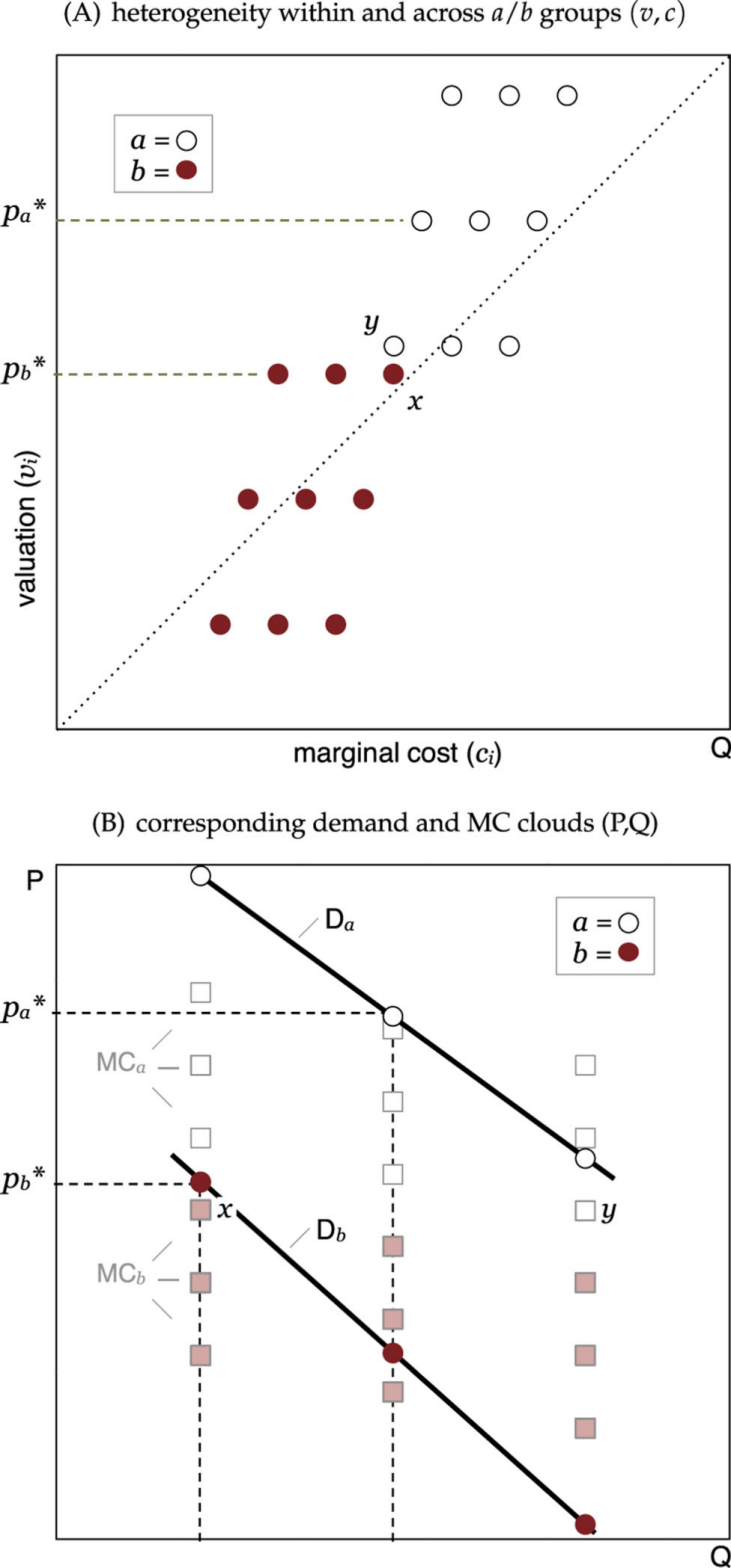
Extension: Demand heterogeneity within and across groups. The figure extends the intuition of Figure 3 to show how optimal prices are affected by allowing for both within- and across-type demand heterogeneity. The within-type demand heterogeneity introduced in the figure implies that within each of the a and b types, consumers facing the same expected costs have differing willingness-to-pay. Across-type demand heterogeneity can be seen by comparing the a (hollow) and b (solid) points, similar to Figure 3. In the corresponding demand diagram of panel B, willingness-to-pay is shown with circles and costs are shown with squares. As in the case where there is only across-type heterogeneity in Figure 3, no single price generates the best feasible allocation. Here, separate pricing along the a/b type leads to more individuals sorted efficiently relative to uniform pricing, even though there remains residual unpriced heterogeneity that rules out a first-best solution. See the notes to Figures 1–3 for additional documentation.

Figure 4 incorporates cost differences across groups and within-group demand dispersion while maintaining the feature of Figure 3 that, on average, there are differences in demand across the two groups. The panels parallel panels A and B of Figure 3, but here valuations across individuals within the a/b types also differ conditional on cost or, equivalently, costs differ conditional on valuation. As above, individuals are represented by points. The top panel is plotted in (v,c) space and includes a 45-degree line that separates efficient from inefficient enrollment. The bottom panel plots the corresponding demand diagram.

As in Figure 3, these assumptions generate different optimal prices for the two types, $p_{a}^{*}$ and $p_{b}^{*}$. In contrast, now the group with the higher willingness-to-pay conditional on cost, type a, optimally faces *higher* prices. Note that costs overlap across groups at exactly one point, where the costs of individuals x (type b) and y (type a) are identical. Conditional on this common cost, types a have higher willingness-to-pay. Thus, allowing for within-group demand dispersion is theoretically important. In particular, it may no longer be the case that the group with higher demand optimally faces lower prices.

Within-group heterogeneity rules out the possibility of a first-best allocation. It nonetheless remains true that price discrimination across the Figure 3, but here valuations across individuals within the a/b types is constrained optimal relative to the best nondiscriminatory price. In the example of Figure 4, price discrimination leads to *more* individuals self-sorting efficiently, even if no prices can sort *all* consumers efficiently.

### 2.7 Interaction with risk adjustment

The textbook solution to Akerlof-style price distortions is consumer subsidies that have the effect of lowering the uniform price that consumers face. Another prominent regulatory response to selection in health insurance markets is risk adjustment.^18^ Risk adjustment works as a nonuniform subsidy to insurers that varies with consumers’ expected cost types, $c_{i}$. It compensates insurers or plans that enroll low expected cost types and taxes insurers or plans that enroll high expected cost types. In terms of the demand diagram in Figure 3, risk adjustment flattens the insurer’s perceived marginal cost curve, net of the risk-adjusted transfers. (Social efficiency nonetheless occurs along the actual marginal cost curve.) With the proper level of risk-adjustment subsidies, the regulator could implement any uniform price as a competitive market equilibrium outcome, just as with direct consumer subsidies.^19^

However, risk adjustment does nothing to address the inefficiency arising from nondiscriminatory prices in a setting with demand heterogeneity. Risk adjustment does not alter the facts that (i) consumers with different demand face the same prices, and (ii) consumers with different demand must face different prices to sort themselves efficiently. Research subsequent to this study, including Layton (2014) and Layton, Ellis, and McGuire (2015), has examined this interplay of risk adjustment and demand heterogeneity.

## 3. Data and reduced-form empirical strategy

### 3.1 Data

I examine consumer plan choices in an employer-sponsored health insurance setting to demonstrate that the kind of demand heterogeneity described in Section 2 is empirically relevant. The employer health plan setting is uniquely well suited for identifying the effects of interest for several reasons. First—unlike other settings, such as the ACA exchanges or other individual markets—one can observe men and women, young and old, sick and healthy, rich and poor, all facing the same menu of insurance options at the same prices. This is because the Employee Retirement Income Security Act of 1974 (ERISA) and the Health Insurance Portability and Accountability Act of 1996 (HIPAA) rule-out discrimination in employee health benefits made on the basis of an employee’s or dependent’s sex, race, age, national origin, religion, or disability, as well as health status or genetic information. Second, the particular plans here are differentiated only in cost sharing—not provider networks or other plan features. This facilitates a straightforward comparison of the plan options in an expected utility framework, which I exploit in Section 5. Finally, the detailed claims data contain all the information needed to calculate unbiased estimates of the insurer’s marginal costs.

Data come from the administrative health insurance records of a large anonymous employer.^20^ Employees in the firm were offered the choice of two vertically differentiated preferred provider organization (PPO) plan options. These plans differed only in their cost-sharing rules—that is, the degree of consumption smoothing they provided. Denote the two contract options L and H for low and high coverage. Compared to L, contract H had a lower deductible ($300 versus $500), lower coinsurance (10% versus 20%), and a lower out-of-pocket maximum ($2300 versus $4250).^21^ Table 1 summarizes the plans.

The employee contributions to plan premiums are not available in the data, so the prices of these plans are not known. In contrast, average plan costs, which are systematically related to premiums in competitive market settings as well as in most employer-subsidized plan settings, can be directly and precisely calculated by aggregating the total claims paid out by each plan.^22^ Fortunately, identifying demand heterogeneity across groups (the test of Equation (4)) only requires observing the plan choices of consumers who face the same options at the same prices. It does not require knowing what those prices are.

The data are composed of service-level claims files and employee enrollment records. Claims include the line-item billing and diagnosis information on all enrollees. This covers every contact with a medical service provider and all prescription drug purchases. Besides prices and quantities, claims records list the portion of each bill paid by the enrollee and by the health plan. Most records include one or more diagnosis or procedure code. The demographic and diagnoses information on patients (δ) combined with plan cost-sharing rules (ϕ) are used to construct a measure of expected insurer payouts that corresponds to the term $\hat{c}(\phi ,\delta )$ in the model in Section 2.

The main estimation sample is limited to the employees who enroll themselves without dependents in one of the employer’s plans.^23^ The claims data span 2004–2007. To avoid the complication of enrollees aging into Medicare and out of the sample over the panel, I cap the sample at age 59. This results in a sample of about 22,000 enrollee-years, composed of 60% males, with an approximately uniform age distribution between 18 and 59. Appendix A.2 gives additional details on worker and firm characteristics.

**FIGURE 5.**
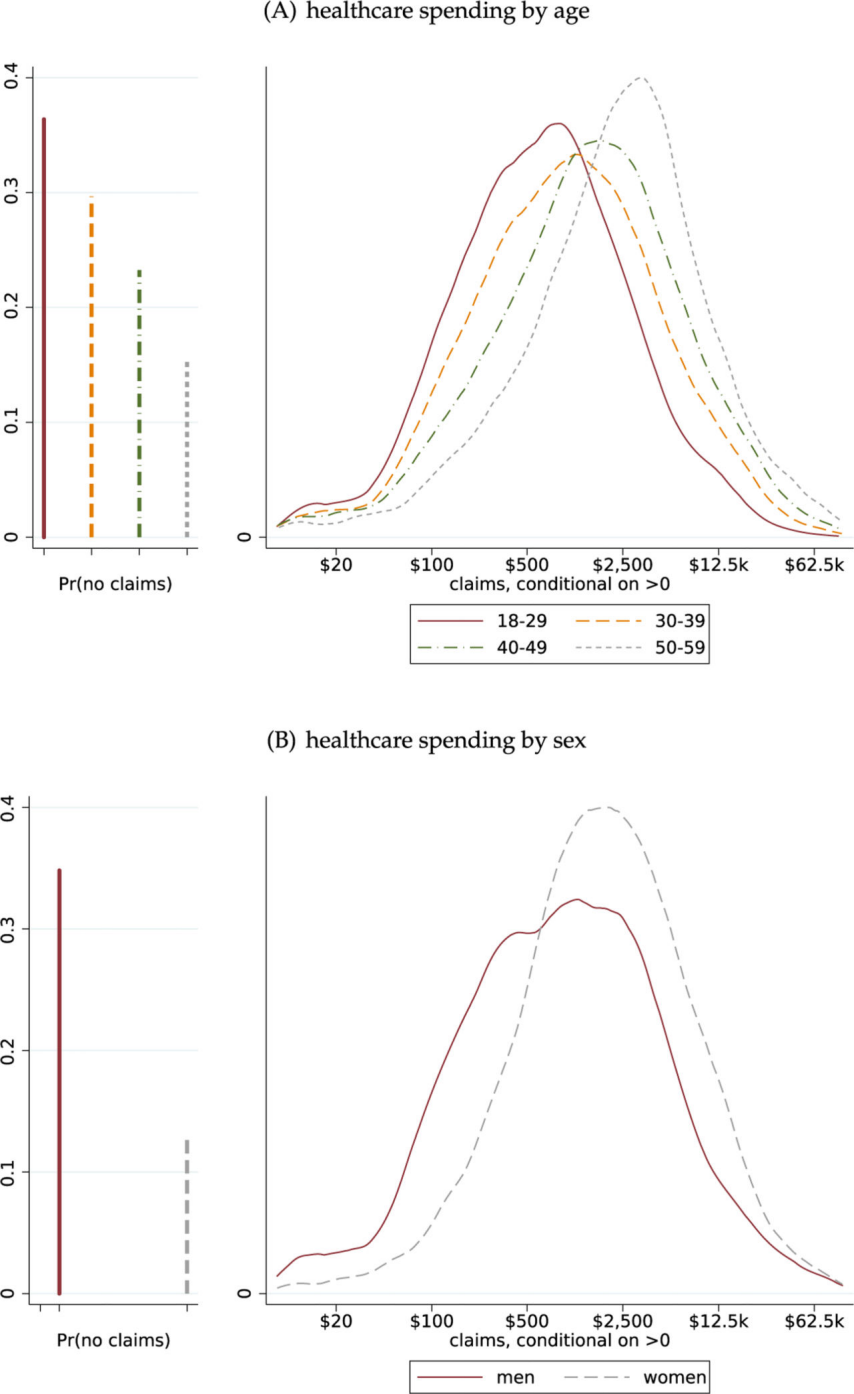
Summary statistics: Distributions of realized healthcare costs. The figure shows the distributions of realized healthcare spending by age (panel A) and by sex (panel B). The left side of each panel indicates the fraction of enrollees with no claims in the plan year; that is, no contacts with a healthcare provider. The right side of each panel plots a kernel density estimate of the total healthcare spending in the plan year (insurer plus consumer shares), conditional on positive spending.

Figure 5 shows the detailed distribution of healthcare spending in the sample by age and sex. The figure plots a kernel density estimate of positive spending and separately indicates the fraction in each subsample without any healthcare utilization. Utilization increases with age and is higher for women than men. The figure shows that within these demographic groups and overall, healthcare spending in the sample follows a mixture distribution that includes a point mass at zero and an approximately log-normal component.

Table 2 provides a high-level summary of employees’ plan choices and healthcare spending. The first column tallies the fraction of enrollees who chose the more generous insurance option, plan H. Healthcare consumption in columns 2–4 is measured as the total bill paid to service providers. Column 2 gives the average overall healthcare expenditure. Columns 3 and 4 display these average healthcare costs conditional on the plan chosen. The first row lists these aggregate statistics for the entire estimation sample, pooling years 2005–2007. Consistent with most studies of selection in health insurance markets, adverse selection is reflected in the plan choices. Enrollees who choose plan H consume twice as much healthcare on average as plan L enrollees.^24^ In the absence of preference heterogeneity, the inefficiency generated by this type of selection can be counteracted with subsidies in a competitive market setting or with properly set uniform premium subsidies in employer plans. Moving down the table, the same general pattern of adverse selection exists within each group as it does in the sample overall. The average level of healthcare costs varies widely across the groups. For instance, the average consumption of a 50- to 59-year-old is around 3.5 times that of an 18- to 29-year-old ($6305 versus $1820).

Importantly, Table 2 shows the first suggestive evidence that there could be significant demand heterogeneity within and across groups. A one-to-one mapping from insurable risk to plan take-up would tend to produce expected costs in plan H that are higher than expected costs in plan L, overall and when comparing these costs across the age groups. In contrast, Table 2 shows that 50- to 59-year-olds who choose the *low* coverage plan generate average costs of about $5000, while 18- to 29-year-olds who choose the *high* coverage plan generate average costs of only $3000. This suggests that consumers are not uniformly following a common decision rule for when to take up H. The summary statistics are merely suggestive on this point. The systematic analysis below compares plan choices between young and old enrollees who face the same ex ante level of expected healthcare costs.

### 3.2 Regression framework and identification

Abbreviating the indicator function notation (from I(z=a) to $I_{a}$) and rearranging terms in (4) yields $E\left[E\left[I_{j}|I_{a}\right]-E\left[I_{j}|I_{b}\right]|c\right]$. This expression for the difference in the conditional expectations naturally maps to a regression framework. The expression is equal to the coefficient on the type indicator $\left(I_{z}\right)$ from an ordinary least squares (OLS) regression of plan choice on types (z) that controls for costs (c).^25^ Thus, the sufficient statistic test in (4) is reduced to examining coefficients on group indicators. Using the claims data, I implement the test by estimating OLS regressions of the form

(5) $$H_{i}=\sum_{z\in Z}\beta_{z}I_{z_{i}}+f\left(c_{i}\right)+\beta_{X}X_{i}+\epsilon_{i},$$

where $H_{i}$ is an indicator for choosing plan H, and $I_{z}$ are indicators for sex and age bins. The coefficients of interest are $\beta_{z}$. The cost variable $c_{i}$ is described more below, and in various specifications is controlled for linearly or nonparametrically. To maximize power, individuals observed in several years of data are included, with standard errors clustered at the person level. Year effects are included in all regressions. Additional controls X are described below.

Irrespective of the control set included, the coefficients of interest, $\beta_{z}$, are not interpreted as causal effects. Several unobservables that are presumably important in the consumer’s decision process (e.g., risk aversion, wealth at risk, the dispersion of risk) would drive correlation between $I_{z_{i}}$ and $\epsilon_{i}$. This poses no problem for the identification strategy. Any correlation between an observable z and demand is evidence of the phenomenon described by the model in Section 2. This is precisely because it indicates that z is a correlate of some unobserved preference or constraint that is generating demand differences.

In this sense, the regression in Equation (5) constitutes a sufficient statistic test: It reveals demand differences without making claims about the underlying primitives that generate the demand. Any utility function primitives or constraints that would generate the same demand curves would have the same implications for welfare.^26^

With that in mind, the only appropriate controls X to include in the regression would be those that could impact contract choice through some channel other than via correlation with the enrollee’s insurance demand. One potential confounder of this type would be if the regression failed to adequately limit the sample to employees facing the same choice set and premiums. Focusing on the employer setting—and in particular, focusing on a single firm offering a common choice set to all enrollees—essentially eliminates the possibility of this type of misspecification. The reason is that the Employee Retirement Income Security Act of 1974 (ERISA) and the Health Insurance Portability and Accountability Act of 1996 (HIPAA) prohibit employers from charging different premium contributions to employees of different gender, race, age, national origin, religion, or disability. ERISA requires that “similarly situated” employees face identical premium contributions. The only theoretically relevant controls are those that might enter into defining workers as “similarly situated.” Therefore, I include indicators for hourly versus salary pay, full-time versus part-time employment, and union status. In practice, adding these controls has essentially zero impact on point estimates and confidence intervals.

### 3.3 Expected cost measure

To control for expected costs, I use the Johns Hopkins Adjusted Clinical Grouper (ACG) to aggregate the rich clinical information contained in the claims files into a predicted expected health spending measure ${\hat{C}}_{i}$. The algorithm predicts period t total spending at the individual level using demographics and t−1 diagnosis and utilization information.^27^ This approach and tool has been used and validated in the recent literature, including in Handel (2013) and Handel, Kolstad, and Spinnewijn (2015). The resulting prediction is for total healthcare consumption in dollars; that is, the total (enrollee plus insurer) payments made to providers. For various parts of the analysis below, I convert predicted health spending, ${\hat{C}}_{i}$, to the insurer’s predicted cost, ${\hat{c}}_{ij}$, by applying plan cost-sharing rules to the total spending amount.

For the sufficient statistic test embodied in Equation (5), ${\hat{c}}_{i}$ need only satisfy two conditions. First it must be an unbiased predictor of insurer costs. Second, controlling for ${\hat{c}}_{i}$ must eliminate any correlation between the observable type and expected costs: $\rho_{c,z|\hat{c}}=0$ The panel nature of the data makes it possible to directly test these conditions by comparing the prediction of expected costs to the ex post realized claims costs. In Section 4, I confirm that a regression of realized costs on predicted costs yields a slope coefficient near 1 and an unconstrained intercept of 0.

An alternative approach to controlling for the expected cost term in Equation (5) would be to condition on realized healthcare spending, which is directly observed in claims. Realized spending represents a noisy measure of expected spending, where the error term is potentially nonclassical because the realization is bounded below at zero. For that reason, the main analysis relies on the ACG prediction, but in several robustness checks below, I show that the revealed choice patterns are similar when conditioned on realized costs.^28^

## 4. RESULTS

In this section, I present the main empirical finding of the paper: that willingness-to-pay for insurance covaries strongly with age and sex, after fully accounting for expected (or realized) healthcare spending differences. Here, the intention is to document patterns of demand that match the model, while imposing minimal economic and econometric assumptions. In Section 6, I test additional predictions of the model and calculate policy counterfactuals by assuming an expected utility framework that imposes more restrictive assumptions.

### 4.1 Main results: Demand patterns across age and sex

**FIGURE 6.**
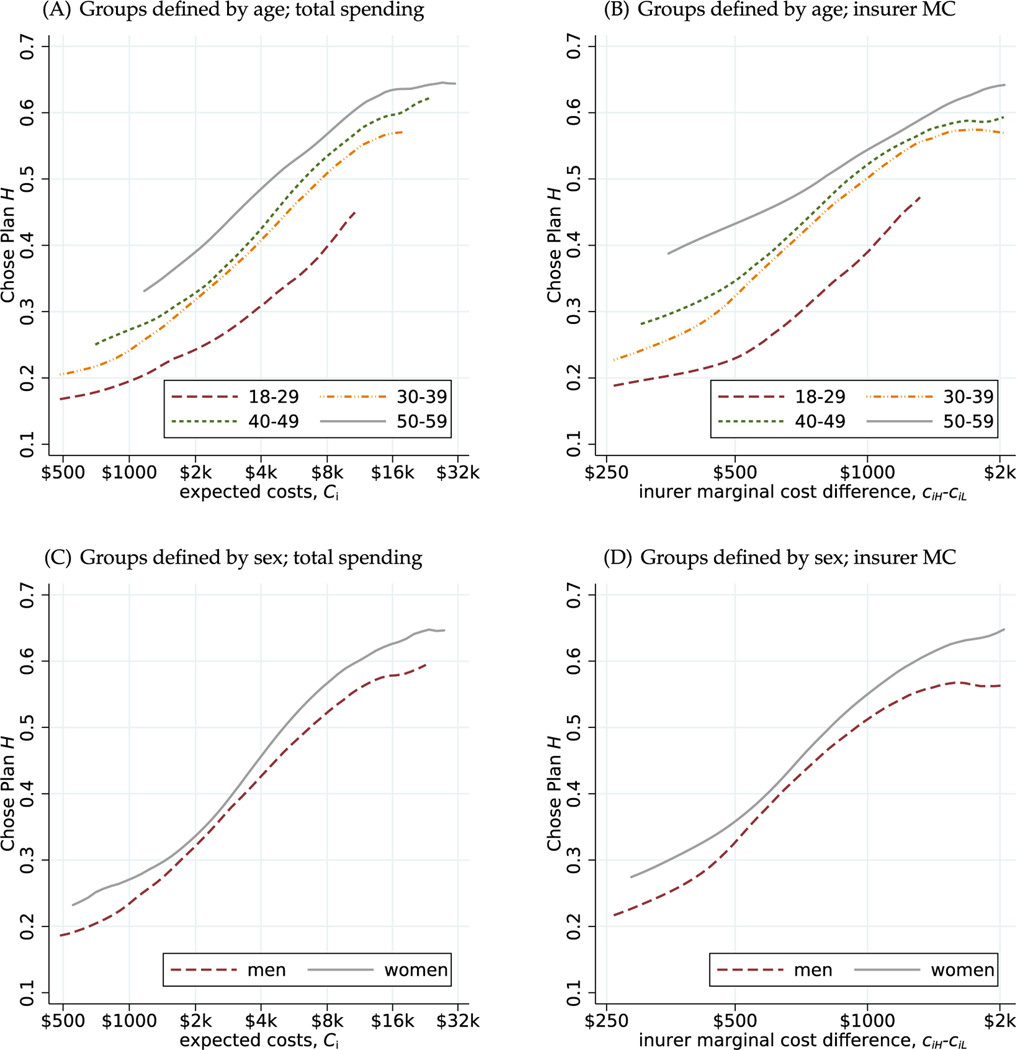
Empirical analogue of Figure 3: Demand heterogeneity across groups. The figure plots local polynomial regressions of plan choice on measures of expected healthcare consumption. In the top panels, the regressions are estimated separately in four age bins. In the bottom panels, the regressions are estimated separately by sex. The conditioning cost variable used in panels A and C (left) is the total expected healthcare consumption, ${\hat{C}}_{i}$. The cost variable used in panels B and D (right) is the insurer’s expected cost of providing plan H minus the insurer’s expected cost of providing plan L to the same individual (${\hat{c}}_{iH}-{\hat{c}}_{iL}$). Horizontal axes are scaled in logs. The regressions underlying these plots correspond to the sufficient statistics test described in Equations (4) and (5).

Figure 6 plots local polynomial regressions of plan choice on expected costs estimated separately by age group in the top panels and by sex in the bottom panels. These plots are semiparametric versions of the linear regression in Equation (5). The dependent variable is an indicator for choosing plan H, which is approximately linear in log expected costs. In panels A and C (left), I use total expected healthcare spending, ${\hat{C}}_{i}$, as the cost control variable. This specification illustrates the relationship between insurance demand and medical risk with minimal data manipulation. In panels B and D (right), I condition instead on the theoretically relevant measure from Equation (4): the difference in expected insurer spending between the plans that is implied by the individual’s total expected healthcare spending, ${\hat{c}}_{iH}-{\hat{c}}_{iL}$.

There is a natural parallel between the empirical results in Figure 6 and the model of Figure 2. Figure 6 plots plan take-up and panel A of Figure 3 plots plan valuations. The take-up rate is just the fraction of consumers for whom willingness-to-pay for plan H over plan $L\left(v_{iH}-v_{iL}\right)$ exceeds the difference in employee premium contributions between these plans. The plots show that conditional on either measure of expected healthcare costs, take-up of H increases monotonically in age. Women also appear to have a higher willingness-to-pay for H. To understand the size of the demand differences implied by the plots, consider holding take-up of plan H fixed at 40% in the top left panel of Figure 6. Take-up rates reach 40% at approximately $2000 of expected spending for 50- to 59-year-olds. But among 18- to 29-year-olds, take-up rates reach 40% only at $8000 of expected spending.

To assess statistical significance, as well as control for other covariates, Table 3 reports results from a series of plan choice regressions. Guided by the log linearity apparent in Figure 6, I regress an indicator for choosing plan H on the natural logs of expected costs. Columns 1–3 evaluate age and sex coefficients in the same regression and add controls for state fixed effects and worker characteristics. These controls are important in principle if workers at different plant locations or with different status (hourly/salary, part time/full time, unionized/not unionized) face different premium contributions. In practice, the controls have almost no impact on coefficients or significance. Columns 4 and 5 evaluate age and sex separately—a relevant separation for considering markets in which regulators allow pricing on age but not sex, or on sex but not age.

Consistent with the semiparametric plots, the regressions in Table 3 confirm there is significant demand heterogeneity that is associated with observables. Coefficients on the age dummies can be interpreted as percentage point differences in take-up rates relative to the excluded category, 18- to 29-year-olds. These estimated coefficients correspond to large differences. Taking parameters from column 3, mean take-up rates among 18- to 29-year-olds are 42% lower (= 0.093/0.220) than among 50- to 59-year-olds who are equally costly to insure. Differences across sex are less pronounced than across age, but the coefficients indicate that women have a stronger preference on average for more comprehensive insurance. The fact that demographic groups differ not only in underlying risk but also in insurance demand conditional on risk is a pattern necessarily missed by studies that have, in the absence of detailed claims data, relied on demographic variables as proxies for health expenditure risk.

It is important to note that age and sex are not assumed to be exogenous in these regressions. These observables are merely correlated with a variety of unobserved parameters and constraints that are contained in the error term. As the analysis in Section 2 demonstrated, this correlation between an observable characteristic and unobserved demand determinants is exactly the pattern of interest because it indicates that such observables can be used by the social planner or regulator to more efficiently price-discriminate. To the extent that the age and sex patterns shown here carry over to other markets like Medicare, Medicaid, and the ACA exchanges, these results are informative of the potential inefficiency of nondiscriminatory pricing regulations in these other markets.

### 4.2 Robustness

Although age and sex need not be uncorrelated with the error term in Equation (5), identification nonetheless requires that these observables have no residual correlation with insurer losses after controlling for ${\hat{C}}_{it}$. A potential disadvantage of using the ACG prediction rather than realized claims to control for expected costs is that the algorithm could in principle introduce bias in expected costs that differed systematically by age.^29^ The panel nature of the data permits a direct test for this type of bias because the prediction ${\hat{C}}_{it}$, which is based on diagnosis and utilization information from the preceding plan year (t−1), can be compared to its realized value, $C_{it}$. It is straightforward to test whether age remains correlated with realized spending after conditioning on ${\hat{C}}_{it}$.

Table 4 displays the results from a series of regressions of realized healthcare spending on the expected spending measure, with and without controls for age.^30^ As a baseline, the regression in column 1 includes all enrollees in the main estimation sample, and ${\hat{C}}_{it}$ is the only regressor. Consistent with unbiased prediction, the slope is very close to 1 and the intercept is close to 0. Columns 2–6 of Table 4 address the question of whether ${\hat{C}}_{it}$ is differentially biased by age. Column 2 adds age bin indicators to the regression. The F -statistic on the additional age variables demonstrates that these have no residual power (p=0.72) to predict realized health spending after conditioning on ${\hat{C}}_{it}$^31^. Columns 3–6 rerun the regression separately by age group. Even in these more flexible specifications, there is no evidence of bias. For each of the subsamples comprising columns 3–6, the slope estimates are close to 1, and the intercepts are economically small and not statistically different from 0.^32^ For completeness, Appendix Figure A2 plots a raw scatter of $C_{it}$ versus ${\hat{C}}_{it}$ separately for each age bin.

Table 4 and Figure A2 provide clear evidence against the possibility that the observed differential demand patterns are spuriously driven by residual correlation between age and plan choice that is not partialed out by controlling for ${\hat{C}}_{it}$ . It is important to note that even if—counter to the findings of Table 4—there were some differential prediction error in ${\hat{C}}_{it}$ across age groups, it would be unlikely to be large enough to spuriously generate the magnitude of the observed demand differences. For example, Figure 6 shows that expected spending estimates would have to be biased by around $6000 between the youngest and the oldest groups (about 400% of the 18- to 29-year-old mean) for unbiased versions of the two nonparametric plots to overlap. If this level of bias were present, one would expect a coefficient on the age 50–59 indicator in column 2 of around this magnitude. In contrast, column 2 shows a precisely estimated zero for this coefficient.

It is also worth noting that moral hazard, or selection on moral hazard (Einav, Finkelstein, Ryan, Schrimpf, and Cullen (2013)), is unlikely to account for the patterns of results in Table 3 and Figure 6. This is because the conditioning variable, ${\hat{C}}_{it}$, is based on t−1 information (primarily demographics and diagnoses) rather than the period t realization of spending that occurs under the chosen plan’s cost-sharing rules. However, if one assumed that moral hazard were a source of bias, it would bias against the observed results: Moral hazard would suggest that the unobserved plan L spending among plan H enrollees is overstated, as cost sharing is lower in H than L.^33^ Because the probability of choosing H increases in age, this would imply that, on average, expected costs are overstated for older consumers relative to younger consumers. In the context of Figure 6, accounting for this would require differentially shifting the curves for the older groups leftward. However, this would have the effect of widening the observed gaps. Thus, if the measure of expected costs were contaminated by moral hazard, it would bias against the main empirical finding.

### 4.3 What drives demand differences?

Uncovering the determinants of the choice patterns revealed in Table 3 is not a main goal of this paper. Nonetheless, the empirical results above prompt the question of why older consumers place such a high valuation on more complete insurance coverage. Is it preference heterogeneity? Is it differences in higher moments of the risk distribution? Is it unobserved (to the econometrician) private information? In this section, I take a first step to investigating this issue. However, it is important to note that under the standard neoclassical assumption that consumer choices reveal consumer preferences, these documented demand differences themselves are sufficient to indicate welfare-improving price discrimination by age. Any differences in utility parameters (e.g., risk attitudes) or in constraints (e.g., wealth) that would generate the same demand differences across groups would have the same welfare implications.

One conceptually straightforward possibility is that, conditional on expected spending, the variance of risk distributions is wider for older enrollees. Mean-preserving risk spreads would lead to different plan choices, even holding fixed both risk preferences and expected spending. Table 5 investigates the possibility, beginning in column 1, by repeating the main result from Table 3 for ease of comparison. The next two columns test the hypothesis that the spread of risk, operationalized as prediction error conditional on the expectation, drives the results. Column 2 controls for the absolute value of the difference between expected and realized costs, $\left|C_{it}-{\hat{C}}_{it}\right|$, and column 3 controls for its square. While, consistent with risk aversion, these variables are significant predictors of choosing H, their inclusion in the regression does not alter the demand patterns by age group relative to column 1. Demand heterogeneity also persists in the structural model of Section 5, where differences in higher moments of risk by age are estimated and fully accounted for.

In columns 4 and 5 of Table 5 and in the top panel of Appendix Figure A3, I examine whether differences in private information on health risk can account for the patterns in the data. The conditioning cost variable in these regressions is the actual ex post healthcare spending, $C_{it}$. This spending variable is realized a year or more after the time at which the plan choice is made.^34^ If the demand differences were to disappear with the inclusion of this control, it might indicate that older and younger consumers differed in take-up because they differed in their ability to forecast healthcare use. To match the specification in column 1 and the apparent log-linearity in Figure A3, I take natural logs of this spending measure. Unlike expected spending, realized annual spending is zero for some enrollees. I take two approaches to handling the zeros. In column 4, I translate the data by adding $100 to the total annual claims for each enrollee. In column 5, I drop the zeros. Both of these ad hoc adjustments yield the same result: Conditioning on realized costs, the differences by age persist. The top panel of Figure A3 plots the semiparametric versions of these regressions, revealing the same patterns. These findings rule out the possibility that some age-specific selection on private information is driving the correlation between age and demand in the main specification.

Another class of explanations is behavioral/psychological. Such explanations could have importantly different welfare implications because these phenomena imply that the principle of revealed preference is invalid, and so standard welfare analysis would likewise be invalidated. The setting here is ideal for identifying demand differences but not for examining the possibility of behavioral phenomena behind them. Nonetheless, to the extent possible in these data, I evaluate the role of two behavioral explanations shown to be important to health plan choice in other work: incorrect subjective assessment of healthcare needs (Abaluck and Gruber (2011)) and inertia (Handel (2013)). Understanding the extent to which demand differences can be explained by such behavioral phenomena is important for optimal pricing. If choice frictions were correlated with the observable demand differences across groups, then such differences in demand would not necessarily be informative of true welfare differences.

There is no linked survey that reports consumers’ self-evaluation of health risk, so a direct comparison of subjective beliefs with objective risks is not feasible. However, one potentially salient data point for consumers may be last period’s spending, which will be an imperfect proxy for future spending. If younger and older consumers have differential behavioral biases toward relying on this type of backward-looking heuristic, it could drive the observed demand differences of column 1, which are conditional on unbiased expected spending. Columns 6 and 7 analyze the possibility, controlling for last period’s realized costs rather than expected costs for the coming plan year. Zeros in the realized spending measure are transformed in column 6 and dropped in column 7. The corresponding semiparametric plots are included in the bottom panel of Appendix Figure A3. The monotonic pattern of demand by age persists, providing some evidence against the possibility that younger and older employees make similar choices conditional on a last-period decision heuristic.^35^

With respect to inertia, the young and old may be differentially active or passive in their annual plan choices, and over time this may lead to systematically different patterns of choice conditional on expected costs. It is also possible that younger and older workers, on average, began their firm tenure during a period when plan options or defaults were different.^36^ Some insight into the importance of inertia and defaults can be gleaned by restricting the analysis to a smaller sample of employees who are entering their first year of health plan enrollment during the data series. In the first year, switching costs are likely to be smaller, the default option will be the same across all enrollees, and younger and older employees are more likely to have the same experience with the firm’s plans.

To investigate, I limit the sample to employees in the first year of enrollment in columns 8 and 9 of Table 5. Expected healthcare consumption cannot be calculated for first-year enrollees since they have no medical history to use as a basis for prediction. Therefore, I use ex post realized consumption from the first year of enrollment as an imperfect control for expected costs. The coefficients show that the demand heterogeneity across age is present even at initial enrollment. The differential enrollment patterns are therefore not likely to be driven by older employees “aging in place,” by older employees entering employment at a time of different default options, or by older employees having more experience with the employer’s health plans.

In sum, the data suggest that the revealed demand differences are not driven by higher moments of expenditure risk, residual private information, backward-looking heuristics, or inertia. Other plausible explanations that are an avenue for future investigation include differences in risk preferences and income. Unfortunately, there is very little scope to differentiate between these alternatives with the data at hand: There are no independent (e.g., survey-based) measures of risk aversion in this administrative data. With respect to income, a constant relative risk aversion (CRRA) model would suggest that older employees, whose income and wealth is unobserved but is likely higher, would have *lower* willingness-to-pay for insurance, deepening the puzzle of the revealed patterns. However, as Mahoney (2015) shows, bankruptcy laws create a floor on financial risk, which in turn affects demand for health insurance. Acknowledging that older workers may have greater value at risk due to bankruptcy protections could reverse the predictions of lower insurance demand for the wealthy without departing from a CRRA (or CARA) expected utility framework. Another possibility related to income is differentially high liquidity constraints for younger workers. An important avenue for future work will be to develop research designs that can disentangle the sources of the demand differences that this paper highlights.

## 5. Demand estimation

Interpreting the reduced-form results of Section 4 through the lens of the model of Section 2 implies that uniform pricing regulations would generate inefficient insurance allocations in this setting. Precise numeric statements about optimal prices and welfare losses require tracing out the demand and marginal cost curves. In this section, I estimate a structural discrete choice model that can be used to trace these curves, with the important caveat that unlike the reduced-form results above, identification here requires assuming a particular functional form of utility.

### 5.1 Discrete choice model

Consider a consumer facing the discrete choice between the two insurance contracts offered by the employer.^37^ The expected utility generated by these two contracts differs because the contracts differ in prices (i.e., employee premium contributions) and because they differ in the out-of-pocket spending risk to which they expose the consumer. In the empirical context of this paper, the choice between plans is potentially well described as a choice over money lotteries: In this setting the plans are distinguished in cost-sharing parameters alone, not in provider networks or in other plan features.

Following the literature, I assume expected utility of the constant absolute risk aversion (CARA) form.^38^ CARA implies that utility over money lotteries is invariant to wealth levels, which is a useful assumption because wealth is not observed in these data. The CARA utility index is

(6) $$u\left(W_{i}-P_{j}-{\text{OOP}}_{ij}\right)=-\frac{1}{\gamma }e^{-\gamma \cdot \left(W_{i}-P_{j}-{\text{OOP}}_{ij}\right)},$$

where γ is the risk aversion parameter. The term in parentheses is the money outcome that agent i faces in each state of the world, conditional on choosing plan j∈L,H. This term consists of initial wealth, W, minus the employee’s contribution to the plan premium, P, and the consumer’s out-of-pocket spending (OOP).

To the basic utility index, I add terms that allow the model to capture differences across age and sex in how plans are valued. Unobserved idiosyncratic tastes that shift the relative valuations of the two plans are represented by $\epsilon_{iH}$, which is a normally distributed, mean zero error term: $\epsilon_{iH}∼N\left(0,\sigma_{e_{H}}^{2}\right)$.

Adding these additional taste parameters and integrating Equation (6) over states of the world yields the expected utility in each plan, $V_{ij}$. The states of the world are defined by healthcare utilization, and the relevant risk distribution is the individual by plan-specific out-of-pocket spending risk $F_{ij}(\text{OOP})$:

(7) $$V_{ij}\equiv E\left[u_{ij}\right]=\int -\frac{1}{\gamma }e^{\gamma \cdot \left(P_{j}+\text{OOP}-\delta_{o}H_{i}-\sum_{a}\delta_{a}I_{a_{i}}\times H_{i}-\delta_{F}I_{F_{i}}\times H_{i}-\epsilon_{iH}\right)}dF_{ij}(\text{OOP}).$$

The vector $I_{a}\times H$ is a set of interactions between a plan H indicator and indicators for the same four age groups defined in Section 4. The vector $I_{F}\times H$ is an interaction between female and plan H. The δ coefficients on these interactions are the estimates of interest, analogous to the age and sex coefficients in the Table 3 regressions. The variables $\delta_{a}$ and $\delta_{F}$ capture preferences for plan H that differ systematically across demographic groups. The term $\delta_{o}$ allows for the possibility that *all* consumers over- or undervalue plan H in a way that cannot be rationalized by adjusting the risk aversion parameter. Because the δ coefficients enter the utility function in the money term, they measure plan preferences in dollars. The error term, as well as the age and sex coefficients, are normalized to zero for plan L, since only differences between the two plans are identified. Likewise, the wealth term $e^{-\gamma \cdot W_{i}}$ that appears in both $V_{iL}$ and $V_{iH}$ can be dropped.^39^

Consumers compare $V_{iL}$ to $V_{iH}$ and choose the plan that yields the highest expected utility, given their characteristics $\left(\delta_{a},\delta_{F}\right)$, idiosyncratic preferences $\left(\epsilon_{H}\right)$, and the out-of-pocket spending risk they face under each plan $\left(F_{ij}(\text{OOP})\right)$. The choice problem aligns closely with the general canonical model described by Equation (1).

### 5.2 Estimation

The full parameter vector to be estimated is $\left[\gamma ,\delta_{o},\delta_{a},\delta_{F},\sigma_{e_{H}}\right]$. An input to the estimation process is the person individual by plan out-of-pocket spending risk distributions. I construct $F_{iH}(\text{OOP})$ and $F_{iL}(\text{OOP})$ in two steps. First, I estimate person-specific distributions of medical utilization risk, $G_{i}(s)$. Then I map these to out-of-pocket spending by mechanically applying each plan’s cost-sharing rules.

To generate $G_{i}(s)$, I begin by creating cells defined by deciles of expected spending separately within each age group. Following Handel (2013), enrollees in the same cell are assumed to face the same ex ante distribution of healthcare risk. Under this assumption, the ex post distribution of observed healthcare consumption within a cell identifies its ex ante distribution. Following a typical parameterization and consistent with the kernel density plots in Figure 5, healthcare utilization is assumed to follow a mixture distribution consisting of a log-normal component (log(u)∼N(μ,σ)) and a point mass at zero of probability weight g.^40^ I estimate the parameters of the mixture distribution (g,μ,σ) separately for each cell via maximum likelihood and then assign these to each individual within the cell. Finally, I convert healthcare utilization at each point in the distribution $G_{i}$ into out-of-pocket costs $F_{ij}$ by applying the cost-sharing rules (deductible, coinsurance, and out-of-pocket maximum) of each plan.^41^

Simulated maximum likelihood (SMLE) is used to estimate the parameter vector.^42^ The procedure, which closely tracks Handel (2013), is described in full detail in Appendix A.3. Intuitively, the process numerically integrates over the enrollee-specific healthcare spending distribution to calculate expected utility in each plan, choosing parameters γ, $\delta_{o}$, $\delta_{a}$, $\delta_{F}$, $\sigma_{e_{H}}$ that maximize the likelihood of generating the observed choice patterns.

### 5.3 Identification

It is important to understand that precision in statements about welfare comes at the cost of imposing the assumptions of CARA utility and correct beliefs about health risk. In particular, in the absence of supply-shock–driven premium variation to trace out demand curves, the expected utility assumption exploits instead the substantial variation across enrollees in the money lottery implied by each health plan. This variation arises from idiosyncratic health spending risk.^43^ In other words, the assumption of an expected utility decision process allows one to infer responses to plan prices (which do not vary in the data) by observing responses to other plan costs (which vary considerably across individuals in the data). This is a strong assumption, and for that reason, the simulations in Section 6 should be viewed as a back-of-the-envelope exercise that extrapolates away from the transparently identified reduced-form results of Section 4.^44^

In contrast, the identification of demand differences across age and sex in the estimation of Equation (7) does not rely on the assumption of an expected utility decision process. Instead, demand differences across demographic groups continue to be identified by the institutional feature that all similarly situated employees face the same prices for the same insurance contracts. The coefficients on the $H\times I_{a}$ and $H\times I_{F}$ interactions pick up effects that are not well described or rationalized by an expected utility framework that assumes agents within these different demographic groups have identical preferences and differ in demand only because of the differences in risk they face.^45^

Finally, plan costs are observed, but employer contributions to plan costs are not, which means that the price difference between contracts L and H that employees face is unknown. Following typical employer premium-setting patterns during this period,^46^ the price difference is assumed to be 20% of the employers’ equilibrium average cost difference of providing plans L and H(=$695).^47^ In estimation, this unobserved price enters identically to the plan H intercept, $\delta_{o}$. Therefore, changing the assumed price difference changes the intercept coefficient dollar-for-dollar but affects no other coefficient estimates.^48^ Intuitively, the $\delta_{o}$ coefficient measures how, conditional on a risk aversion parameter and objective risk, all consumers over- or undervalue contract H relative to its price. As the assumed price changes, $\delta_{o}$ offsets it exactly.

### 5.4 Parameter estimates

Table 6 lists estimated parameter values. Willingness-to-pay for plan H increases monotonically in age. The coefficients indicate that, after controlling for objective risk, 30- to 39-year-olds value the fuller insurance option by $261 more than 18- to 29-year-olds; 40- to 49-year-olds value it by $323 more; 50- to 59-year-olds value it by $508 more. Willingness-to-pay is also somewhat higher among women.^49^ These patterns track the reduced-form results of Section 4. The estimated standard deviation of idiosyncratic tastes ($1351) rationalizes the observation that, within age and sex groups, many consumers with high expected spending choose the lower coverage plan and many with low expected spending choose the higher coverage plan.^50^ In terms of the model, this idiosyncratic taste parameter corresponds to the within-group heterogeneity of Figure 4. Appendix Figure A4 shows that the estimated model fits the data well, replicating the observed demand patterns of Figure 6.

The coefficient of absolute risk aversion is 6.96 · 10^−4^. To facilitate interpretation, Table 7 compares this estimate to risk aversion estimates from other studies. Risk preferences are likely to be context-dependent, and several of the estimates listed in Table 7 are derived from other insurance markets. Nonetheless, the estimate here falls squarely within the range of the existing literature. My estimate of γ comes closest to estimates from Handel (2013), which were also derived in a health plan context, though with a different identification strategy. To further aid interpretation, the last column in Table 7 displays a certainty equivalent measure of the risk parameters. The certainty equivalent here is the amount X that would make someone just indifferent between accepting a gamble in which they win $100 or lose X with equal probability versus a status quo where nothing happens. The certainty equivalent value implied by the estimate of risk aversion here is $93.

## 6. POLICY SIMULATIONS AND WELFARE

In this section, I leverage the structure of Section 5 to (i) demonstrate that the empirical within- and across-group variation in demand corresponds to the model of Figure 4, (ii) compare the optimal uniform price with the optimal age- and sex-specific prices, and (iii) generate back-of-the envelope welfare estimates under a policy counterfactual of optimal age- and sex-specific prices.

### 6.1 Simulation

**FIGURE 7.**
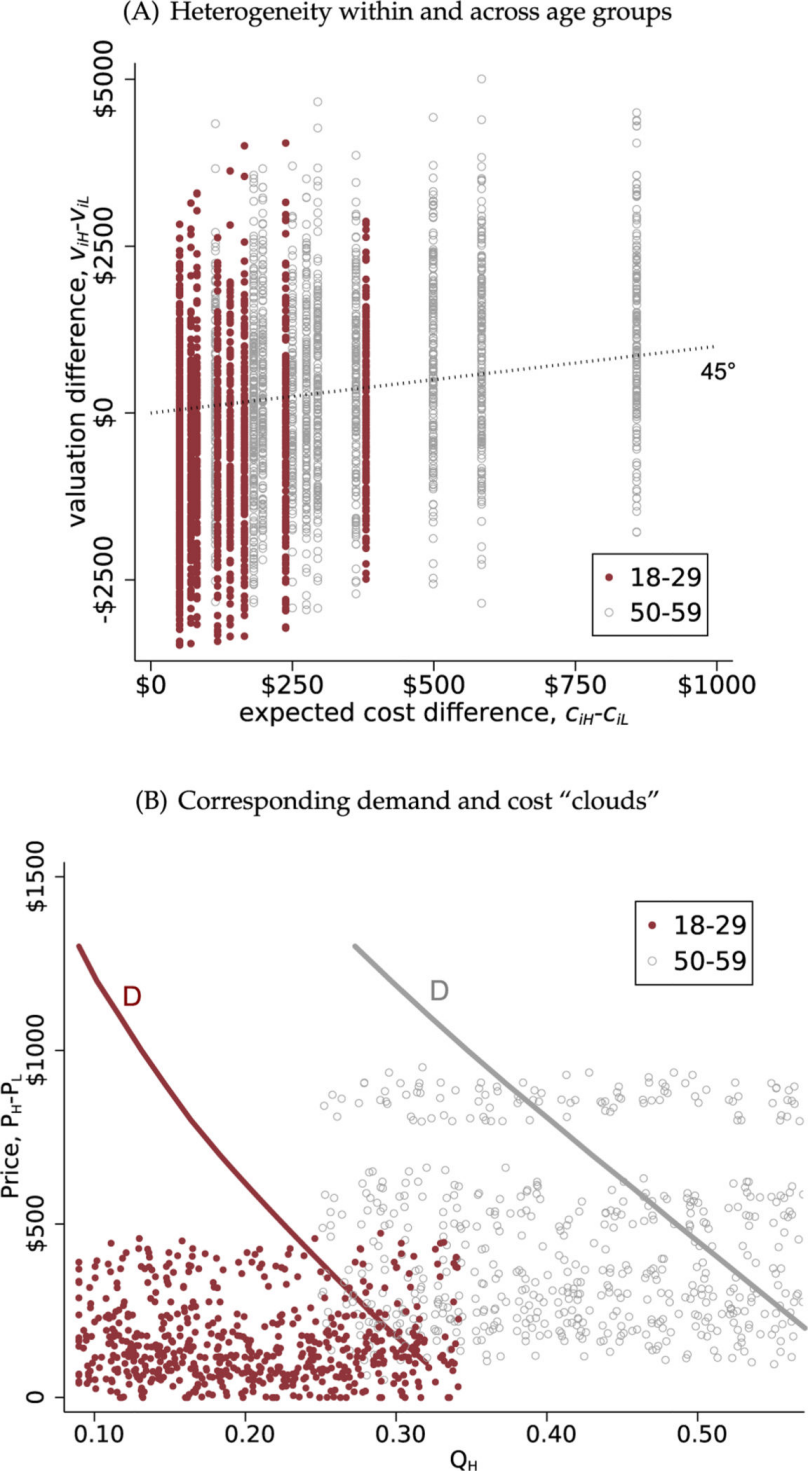
Empirical analogue of Figure 4: Within- and across-group demand heterogeneity. The figure plots the empirical analogue of Figure 4. In panel A, within- and across-type demand heterogeneity is represented in (v,c) space. The dashed 45-degree line separates the cases in which enrolling in H is socially efficient from those in which it is efficient to enroll in L. In panel B, the corresponding demand and average cost clouds are plotted in (P,Q) space. In both panels, the insurer-borne healthcare costs, $c_{ij}$, are calculated directly from the medical risk distributions and cost-sharing rules, as described in Section 3.3. Consumer plan valuations are calculated using the utility function parameters from Table 6 to generate the certainty equivalent value of each plan for each consumer. Because the choice in this setting is between two plans (greater or lesser insurance), the relevant valuations and costs are the differences in valuations and costs between these plans: $v_{iH}-v_{iL}$ and $c_{iH}-c_{iL}$. See Section 6.1 for additional details.

I begin by simulating demand in a way that allows me to complete the link between the model and the data. Figure 7 plots the empirical analogue to the graphical model in Figure 4. To reduce clutter, I focus on comparing the 50- to 59-year-olds and the 18- to 29-year-olds, which are analogous to the a/b types in Figure 4. The insurer portion of expected healthcare costs, $c_{ij}$, are the relevant cost variables here. These are derived directly from the medical risk distributions and cost-sharing rules, as described in Sections 3.3 and 5.2. Consumer valuations are found by applying the utility function parameters from Table 6 to the out-of-pocket spending distributions implied by plan rules and medical risk. These plan valuations are measured in dollars as the certainty equivalent, $CE_{ij}$, that makes agent i just indifferent between paying that sure amount and facing the uncertain loss associated with insurance plan j.^51^ For panel A only, each individual in the sample is given a draw from the distribution of $\epsilon_{H}$.^52^

Panel A of Figure 7 plots plan valuations versus costs. Each scatter point represents an observation. Because the choice in this setting is between two plans (greater or lesser insurance), the relevant valuations and costs are the differences in valuations and costs betweens plans: ${\text{CE}}_{iH}-{\text{CE}}_{iL}$ and $c_{iH}-c_{iL}$. The 45-degree line is plotted for reference. Only for the cases in which ${\text{CE}}_{iH}-{\text{CE}}_{iL}$ exceeds $c_{iH}-c_{iL}$ is the choice of plan H socially efficient.^53^

Panel B of Figure 7 plots the demand curves and cost “clouds” that correspond to panel A. To simulate demand, I calculate the person-specific probability of enrollment in plan H at each price level, denoted $D_{i}\left(P_{H}\right)$. As above, $P_{H}$ is the premium difference between L and H. Demand for H is calculated as the indicator function for taking it up, integrated over the taste distribution:

(8) $$D_{i}\left(P_{H}\right)=\int 1\left(V_{iH}>V_{iL}|\epsilon ,P_{H}\right)\cdot dF\left(\epsilon_{H}\right).$$

This take-up is summed over individuals to generate the demand curve. Insurer expected cost clouds are plotted along with demand. To keep the plot readable, I sample 50 cost points $\left(c_{iH}-c_{iL}\right)$ for each price level on the demand curve, using sampling probabilities equal to $D_{i}\left(P_{H}\right)$. This generates a cloud corresponding to the average cost curve. I also “jitter” the points because many of the expected cost differences overlap. The level of the cost curve is low, and its slope is shallow because the insurer’s spending difference between plans is only about 10% of spending—the difference in coinsurance rates.

Figure 7 closely parallels the model of Figure 4. In panel A of Figure 7, valuation–cost pairs are dispersed within and across groups. The scatter points span the 45-degree line differentially for the two age groups, exactly as in panel A of Figure 4. The residual within-group heterogeneity apparent in the figure highlights that price discrimination on age represents a feasible welfare improvement, but not a first-best, as there is significant heterogeneity that is not (and possibly cannot be) priced. Panel B of Figure 7 likewise mirrors panel B of Figure 4, showing within- and across-group demand heterogeneity, reflected in distinct demand curves for the two age groups and in the cost clouds. This feature that costs do not trace a single line is special to selection markets, where the costs are a function not of some producers’ production technology, but are rather a function of the buyers’ cost types.

With respect to optimal prices, the demand–cost intercepts for the two age groups will clearly differ. Older consumers in this market optimally face higher prices and demand more insurance at those higher prices, while younger consumers optimally face lower prices and demand less insurance at those lower prices. This matches the model in Figure 4, but stands in stark contrast to conventional wisdom, which would imply that young and old would differ in demand only because they differed in insurable risk, and which would predict that take-up conditional on insurable risk would be identical across the two groups.^54^

### 6.2 Optimal pricing counterfactuals

I next turn to optimal pricing and welfare. The welfare estimates are, of course, specific to the empirical setting, so at the end of this section I discuss briefly how the welfare costs of nondiscrimination regulations might differ in other insurance market contexts.

Social surplus is defined as the certainty equivalent valuation of the chosen plan minus the insurer’s cost of providing coverage in that plan. Optimal prices are those that maximize social surplus by inducing consumers to choose the plan that generates the greatest utility net of costs.^55^ The constrained optimal uniform price is found at the intersection of demand and marginal cost in the market overall, while the constrained optimal group-specific prices are found at the corresponding group-specific intersection. I consider these optimal pricing policies, rather than equilibrium pricing, to focus on the novel result of the paper: that optimal pricing as a function of observables generates a feasible welfare improvement over optimal uniform pricing. Nonetheless, all of the prices considered could be implemented as a competitive market equilibrium by a regulator setting appropriate consumer subsidies or tax credits.^56^

Panel A of Table 8 describes a baseline case in premiums equal to 20% of the average insurer cost difference between plans in the observed equilibrium ($695). This level of subsidization is typical for a large employer, but it could also result from imperfect risk adjustment in a competitive market setting. In the remaining panels, prices, take-up rates, and welfare gains under counterfactuals are compared to this baseline. Panel B implements constrained optimal uniform pricing and panels C and D implement constrained optimal pricing by age and sex, respectively. Throughout all the counterfactuals, only prices—not the menu of options—are assumed to change, so that only take-up and costs respond in equilibrium.

In panels B–D, columns 1 and 2 report the counterfactual price and the quantity at that price, columns 3 and 4 report the changes in price and quantity relative to baseline, and column 5 reports the corresponding change in social surplus. This social surplus measure incorporates both buyer’s and seller’s welfare. These differences from baseline are presented overall, as well as within age and sex groups to gauge incidence.

Consistent with the model in Section 2, the reduced-form evidence in Section 4, and as suggested by the simulation displayed in Figure 7, Table 8 shows that there is a feasible welfare improvement in allowing price discrimination by age or sex, even relative to constrained optimal uniform prices. These can be seen by comparing prices in panel B versus C or B versus D. The youngest group of consumers optimally faces prices that are $205 less than those optimally faced by the oldest group in the data. Additionally, women optimally face a price that is $100 larger than the price that optimally sorts men. This implies that the price that sorts men efficiently will lead to women being overinsured, and the price that sorts women efficiently will lead to men being underinsured. If—counter to fact, but consistent with the standard assumption implicit in the literature—these groups had identical demand conditional on objective risk, then no improvement would be feasible relative to the optimal uniform price. The fact that optimal prices differ is because demand (conditional on costs) differs across the demographic groups.

Moving from panel A to B, the resulting gains from optimal uniform pricing, $24/person on average over the population, are comparable in size to other estimates in the literature of the welfare loss of observed pricing relative to optimal uniform pricing in employer plans (Carlin and Town (2008), Einav, Finkelstein, and Cullen (2010), Bundorf, Levin, and Mahoney (2012)). Moving from panel B to C or from B to D, the incremental welfare gains of adopting optimal group-specific prices are an order of magnitude smaller.

The welfare gains associated with implementing group-specific prices relative to optimal uniform prices may be small here because the difference in financial insurance between the two plan options considered here is minor. This leads to optimal uniform and optimal group-specific prices that are on the order of a few hundred dollars apart. To gain some intuition for the result, note that a reasonable approximation of the welfare loss of suboptimal pricing is $-\frac{1}{2}\cdot \left(Q^{*}-Q\right)\cdot \left(P^{*}-P\right)$, where $\left(Q^{*},P^{*}\right)$ are the optimal price and quantity. Comparing panels B and C, enrollment for the 18- to 29-year-olds increases by 2.7 percentage points (11%) and prices decline by $110. Therefore, it is unsurprising to observe average welfare gains of optimally price-discriminating on the order of $1.50 ($\approx \frac{1}{2}\cdot \text{\$}110\cdot 0.027$). Intuitively, the potential welfare losses in this market are limited by the assumption that the consumer must choose either plan L or plan H, either of which provides generous financial insurance.

By the same logic, the welfare losses could be considerably higher in a setting in which remaining uninsured were a relevant outside option. To see this, consider that the average of the marginal cost difference between the plans $c_{iH}-c_{iL}$ for the youngest and oldest groups are, respectively, $\text{\$}134\;\left(=E\left[c_{iH}-c_{iL}|\text{age}\;18-29\right]\right)$ and $\text{\$}360\;(=E\left[c_{iH}-c_{iL}|\text{age}\;50-59\right])$. These averages are not far from the optimal prices, which must lie somewhere along the marginal cost clouds. Now consider a hypothetical choice between plan H and no insurance. Here the marginal cost difference would be $\text{\$}743\;(=E\left[c_{iH}-0|\text{age}\;18-29\right])$ for the younger group and $\text{\$}3850\;\left(=E\left[c_{iH}-0|\text{age}50-59\right]\right)$ for the older group. If these averages were likewise not far from the optimal prices, then it is plausible that these prices could differ by around $3000. This level of difference, which is related to the height of the welfare loss triangle, could imply significantly greater welfare loss from uniform pricing.^57^ In any case, it is important to at least note that the magnitude of the welfare losses in Table 8 are completely context-specific. The general lesson is that demand heterogeneity across identifiable groups implies a feasible improvement by pricing separately.

### 6.3 Equity-efficiency tension

For decades in the United States, employers have been barred by ERISA and HIPAA from setting premium contributions for employee health plans that differ on the basis of age, sex, or almost any other observable worker characteristic. Similarly, in publicly subsidized settings like the Medicare Advantage market, insurers have been required to offer their plans to all eligible beneficiaries at identical prices within a local market, without regard to consumer characteristics like age, sex, and—except for Medicaid eligibility—wealth or income. Most recently, the ACA has placed binding federal restrictions on price discrimination in individual insurance markets. Such regulations are typically framed as addressing an issue of fairness or equity. For example, the Secretary of Health and Human Services describes the ACA’s prohibition against price discrimination on sex as “a key step toward realizing equity within our health care system.”^58^

Absent demand heterogeneity, such nondiscrimination policies aimed at equity would carry no efficiency cost if paired with proper subsidies. However, the empirical exercise above demonstrates that such heterogeneity does exist and can be substantial. In the reduced-form analysis above, the observed take-up of plan H differed by more than 40% between the youngest and oldest consumers facing the same expected losses. Under the expected utility structure of Sections 5 and 6, these patterns implied optimal incremental prices for H that differed by approximately 2.4 to 1 comparing the oldest and youngest consumer age groups and by 1.5 to 1 comparing women and men. Therefore, even with mechanisms like risk adjustment, reinsurance, and nondiscriminatory tax subsidies, the only way to induce consumers to sort themselves efficiently involves exposing them to unequal prices, in violation of the equity goal.

It is interesting to interpret the pattern of optimal age-specific prices found here in light of the ACA pricing restrictions. In the exchanges, price discrimination on age is allowed up to a federal maximum ratio of 3 : 1. Further, states are permitted to enforce narrower age bands than the federal maximum. In 2016, Massachusetts imposed a maximum 2 : 1 ratio, and New York and Vermont prohibited age rating entirely. The range of optimal prices by age estimated here is not directly comparable, most notably because optimal pricing depends on the choice set and the generosity of the contracts, as these affect incremental marginal costs. The menus of plans available in the exchanges differ from the two relatively generous PPOs studied here. Nonetheless, I find that optimal prices follow a schedule that is increasing in age, matching in a superficial sense the pattern of regulated pricing in place in most state exchanges and conflicting with the less common policy of banning age rating. The *prima facie* similarity of the ACA’s federal age banding to an optimally set schedule is probably coincidental, as the age-varying price schedule implemented by the federal rule was likely motivated by the desired extent of implicit transfers from younger to older consumers, rather than by efficiency concerns.

Finally, it is interesting to note that unlike other forms of price discrimination, pricing based on either age or gender—although in direct conflict with the equity objective of nondiscriminatory pricing—does not expose consumers to dynamic risk. Pricing based on health state, in contrast, introduces a new dynamic risk for consumers whose health may deteriorate, as discussed in depth by Handel, Hendel, and Whinston (2015). Age- and gender-based pricing sidesteps issues related to such reclassification risk because there is no dynamic uncertainty related to either.

## 7. CONCLUSION

The conventional wisdom holds that with the proper tools, such as risk adjustment and consumer subsidies, regulators can undo the selection problem created by nondiscrimination policies and generate the best feasible allocation. Building on a recent literature exploring demand heterogeneity, I show here that uniform prices cannot generate the best feasible allocation when there is heterogeneous demand, highlighting an equity-efficiency tension. This equity-efficiency trade-off is a fundamental feature of insurance markets with demand heterogeneity, and there is little reason to assume that demand heterogeneity is not an important part of most insurance market settings. While regulators and policymakers may nonetheless wish to pursue equity objectives, there is an unavoidable efficiency cost of doing so.

There is an important role for future work to uncover what drives the striking demand differences by age and sex uncovered here. As a first step toward that investigation, I show that demand heterogeneity appears not to be driven by differences in higher moments of the risk distribution, by differential inertia across young and old or men and women, by subject beliefs based on last-period heuristics, or by differential private information about future healthcare costs.

Another important task for future work will be to apply the ideas here to other observable consumer types—for example, income-based pricing as in Glazer and McGuire (2011) or prices linked to the dispersion of risk consumers face. Perhaps most importantly, future work should consider how acknowledging demand heterogeneity affects optimal regulatory policy in other markets, particularly markets in which remaining uninsured is a relevant potential outcome. In such settings, welfare losses arising from uniform pricing could be significant.

## Supplementary Material

Supp Material

## Figures and Tables

**TABLE 1.** Cost-sharing rules for the two plans available to employees.

	PPO Plan L	PPO Plan H
	(1)	(2)

Deductible	$500	$300
Coinsurance	20%	10%
Out of pocket maximum	$4250	$2300
Copays		
Emergency department	$0	$0
Well visit	$0	$0
Drugs (brand/generic 30 day)	$10/5	$10/5

*Note*: The table lists cost-sharing rules for the two plans in employees’ choice set. Both plans were PPOs with identical provider networks and differed only in cost-sharing rules. Plan H provides fuller financial insurance. The coinsurance rate is the marginal price faced by the consumer once the deductible has been met. Both the deductible and the coinsurance are counted toward the out-of-pocket maximum.

**TABLE 2.** Summary statistics: Plan choices and average claims costs by group.

	Fraction	Average Healthcare Costs			
	Choosing		Conditional	Conditional	
	Plan H	All Enrollees	on Plan L	on Plan H	Obs.
	(1)	(2)	(3)	(4)	(5)

Full sample	0.35	$3865	$2672	$6090	22,299
By age					
18–29	0.22	$1820	$1460	$3094	4570
30–39	0.31	$2760	$2103	$4239	5787
40–49	0.37	$4355	$2801	$6957	6875
50–59	0.48	$6305	$4958	$7773	5067
By sex					
Men	0.31	$3121	$2193	$5149	13,550
Women	0.40	$5017	$3525	$7223	8749

*Note*: The table presents summary statistics on employees’ plan choices and healthcare consumption for the main estimation sample. The sample consists of employees who enroll in either plan L or H. Column 1 lists the fraction of enrollees who choose plan H. Average costs of healthcare consumed, measured as the total bills paid to service providers, are listed in columns 2–4. Column 2 lists the average expenditure overall. Columns 3 and 4 list expenditure conditional on the plan chosen. The first row lists these statistics for the entire estimation sample. The remaining rows repeat these statistics for different demographic subgroups.

**TABLE 3.** Main result: Demand heterogeneity conditional on expected costs, ${\hat{C}}_{i}$.

Dependent Variable:	Chose Plan H				
Sample:	Full	Full	Full	Full	Full
	(1)	(2)	(3)	(4)	(5)

Expected cost, ln(${\hat{C}}_{t}$)	0.12 (0.01)	0.13 (0.01)	0.13 (0.01)	0.14 (0.01)	0.15 (0.01)
Age 30–39	0.05 (0.01)	0.05 (0.01)	0.05 (0.01)	0.05 (0.01)	
Age 40–49	0.06 (0.01)	0.05 (0.01)	0.05 (0.01)	0.05 (0.01)	
Age 50–59	0.10 (0.01)	0.10 (0.02)	0.09 (0.02)	0.09 (0.02)	
Female	0.02 (0.01)	0.02 (0.01)	0.03 (0.01)		0.03 (0.01)
State FEs		×	×	×	×
Worker characteristics			×	×	×
Mean of dependent variable	0.35	0.35	0.35	0.35	0.35
Observations	22,299	22,299	22,299	22,299	22,299

*Note*: The table reports results from a series of plan choice regressions that parallel the semiparametric plots of Figure 6. These regressions correspond to the sufficient statistics test described in Equations (4) and (5). The dependent variable is an indicator for choosing the fuller insurance option, plan H. All regressions control for expected healthcare spending by including the natural log of the person-specific expected spending, which is predicted using the prior year’s diagnoses and utilization. See Section 3 for full detail. Column 1 includes no additional controls. Columns 2–5 add state fixed effects and worker characteristics, which include indicators for hourly/salary, part time/full time, and union status. Ages 18–29 is the excluded age category. Standard errors (given in parentheses) are clustered at the person level. The acronym FE denotes fixed errors.

**TABLE 4.** Robustness: No correlation between age and realized costs, conditional on ${\hat{C}}_{i}$.

Dependent Variable:	Realized Costs, $C_{t}$					
Sample:	Full	Full	Age 18–29	Age 30–39	Age 40–49	Age 50–59
	(1)	(2)	(3)	(4)	(5)	(6)

Expected costs, ${\hat{C}}_{t}$	1.13 (0.07)	1.12 (0.08)	1.29 (0.23)	0.98 (0.11)	1.14 (0.11)	1.13 (0.14)
Intercept	$8 (201)	−$108 (151)	−$394 (318)	$290 (234)	$114 (311)	−$3 (693)
Age 30 to 39		$30 (182)				
Age 40 to 49		$274 (261)				
Age 50 to 59		$166 (344)				
F-stat on age variables		0.45 *p* = 0.72				
Observations	22,299	22,299	4570	5787	6875	5067

*Note*: The table reports results from a series of regressions of realized medical expenditure on predicted medical expenditure for the same plan year. These regressions test whether age has residual power to predict realized healthcare expenses after the expected cost measure for total spending is included as a control. Column 1 includes the single regressor indicated. Column 2 adds the same age and sex indicators as in Table 3. The group 18–29 is the excluded age category. Columns 3–6 run the regression in column 1 separately within subsamples defined by age group. Unbiased prediction would imply a slope not different from 1.0 and an intercept not different from 0. Standard errors in parentheses are clustered at the person level.

**TABLE 5.** Mechanisms: Private information, heuristics, inertia, and higher moments of risk distribution.

Dependent Variable:				Chose Plan H					
Hypothesis Tested:	Main: Table 3 Col 3	Variance of Risk Distribution		Private Info/Foresight: Realized Costs in t		Look-Back Heuristic: Realized Costs in t−1		Inertia: New Enrollees Only	
Sample:	Full Sample	Full Sample	Full Sample	Full Sample	$C_{t-1}>0$	Full Sample	$C_{t}>0$	Full Sample	$C_{t}>0$
	(1)	(2)	(3)	(4)	(5)	(6)	(7)	(8)	(9)

Expected cost, ln(${\hat{C}}_{t}$)	0.13 (0.01)	0.13 (0.01)	0.13 (0.01)						
Realized cost, ln($C_{t}$)				0.06 (0.00)	0.06 (0.00)			0.05 (0.00)	0.06 (0.00)
Last period realized cost, ln($C_{t-1}$)						0.06 (0.00)	0.07 (0.00)		
Age 30–39	0.05 (0.01)	0.05 (0.01)	0.05 (0.01)	0.07 (0.01)	0.06 (0.01)	0.07 (0.01)	0.06 (0.01)	0.09 (0.01)	0.08 (0.01)
Age 40–49	0.05 (0.01)	0.05 (0.01)	0.05 (0.01)	0.11 (0.01)	0.10 (0.01)	0.11 (0.01)	0.10 (0.01)	0.12 (0.02)	0.10 (0.01)
Age 50–59	0.09 (0.02)	0.09 (0.02)	0.09 (0.02)	0.19 (0.02)	0.18 (0.01)	0.18 (0.02)	0.17 (0.01)	0.12 (0.02)	0.12 (0.02)
Prediction error, $\left|C_{t}-{\hat{C}}_{t}\right|\times 10^{-7}$		125 (033)							
Error squared, ${\left(C_{t}-{\hat{C}}_{t}\right)}^{2}\times 10^{-13}$			859 (385)						
Sex	×	×	×	×	×	×	×	×	×
State FEs	×	×	×	×	×	×	×	×	×
Worker characteristics	×	×	×	×	×	×	×	×	×
Mean of dependent variable	0.35	0.35	0.35	0.37	0.35	0.38	0.35	0.26	0.25
Observations	22,299	22,299	22,299	18,764	22,299	17,809	22,299	7012	8145

*Note*: This table reports results from a series of plan choice regressions. The dependent variable across all columns is an indicator for choosing the fuller insurance option, plan H. The sample, regressors of interest, and hypothesis to be tested vary across columns as indicated. All regressions control for some measure of expected or realized healthcare spending. Observations with zero spending are transformed in columns 4, 6, and 8 to allow taking natural logs. Observations with zero spending are dropped in columns 5, 7, and 9. All regressions control for state fixed effects and worker characteristics, which include indicators for hourly/salary, part time/full time, and union status. Group 18–29 is the excluded age category. Standard errors in parentheses are clustered at the person level.

**TABLE 6.** SMLE parameter estimates from expected utility model.

Parameter	Coefficient	SE

Age 30–39	$261	($71)
Age 40–49	$323	($75)
Age 50–59	$508	($92)
Female	$178	($50)
Plan H intercept	−$832	($153)
Coefficient of absolute risk aversion	0.000696	(0.000101)
Standard deviation of ϵ	$1351	($165)

*Note*: Results from the expected utility plan choice model. Standard errors (SE) are calculated from the inverse of the numerically computed Hessian. See Section 5 for full details.

**TABLE 7.** Risk aversion estimate in context.

Reference	Absolute Risk Aversion	Certainty Equivalent

Cohen–Einav (2007) median	3.4 × 10^−5^	99.7
Metrick (1995)	6.6 × 10^−5^	99.3
Gertner (1993)	3.1 × 10^−4^	97.0
Handel (2010) median	3.7 × 10^−4^	95.2
**This Paper**	**6.96 × 10^−4^**	**93.5**
Handel (2010) mean	7.9 × 10^−4^	92.6
Sydnor (2006)	2.0 × 10^−3^	83.3
Cohen–Einav (2007) mean	3.1 × 10^−3^	76.5
Holt and Laury (2002)	3.2 × 10^−2^	21.0

*Note*: This table compares the estimated coefficient of absolute risk aversion from Table 6 to the literature. To aid interpretation, column 3 displays a certainty equivalent measure of the risk parameters. The certainty equivalent here is the amount X that would make someone indifferent between accepting a gamble in which they win $100 or lose $X with equal probability versus a status quo where nothing happens.

**TABLE 8.** Counterfactual pricing and welfare.

			Relative to Baseline		
	Price $\left(P_{H}-P_{L}\right)$	Takeup of H	Price Change	Takeup Change	Welfare Change
	(1)	(2)	(3)	(4)	(5)

		Panel A: Baseline Case			
Overall	$695	0.311			
18–29	$695	0.184			
30–39	$695	0.279			
40–49	$695	0.338			
50–59	$695	0.430			
Men	$695	0.274			
Women	$695	0.376			
		Panel B: Optimal Uniform Pricing			
Overall	$255	0.427	−$440	0.116	$24.57
18–29	$255	0.282	−$440	0.098	$31.66
30–39	$255	0.394	−$440	0.114	$29.02
40–49	$255	0.459	−$440	0.121	$22.90
50–59	$255	0.556	−$440	0.126	$14.98
Men	$255	0.386	−$440	0.112	$27.57
Women	$255	0.497	−$440	0.121	$19.34
		Panel C: Optimal Age-Specific Pricing			
Overall		0.428		0.117	$25.34
18–29	$145	0.309	−$550	0.125	$33.22
30–39	$220	0.404	−$475	0.124	$29.44
40–49	$270	0.455	−$425	0.117	$23.00
50–59	$350	0.529	−$345	0.099	$16.36
		Panel D: Optimal Sex-Specific Pricing			
Overall		0.429		0.118	$24.86
Men	$210	0.398	−$485	0.124	$27.76
Women	$310	0.482	−$385	0.106	$19.80

*Note*: The table reports plan enrollment and welfare under several counterfactual pricing scenarios. Panel A of Table 8 describes the baseline case of uniform premiums equal to 80% of the average insurer cost difference between plans. Panel B sets the single, nondiscriminatory price for all enrollees that maximizes overall welfare subject to the constraint of a uniform price. Panel C sets the optimal age-specific prices. Panel D sets the optimal sex-specific prices. Columns 1 and 2 report the counterfactual price and quantity at that price. Columns 3 and 4 report the changes in price and quantity relative to the baseline in panel A. Column 5 reports the corresponding welfare change.

## Footnotes

1 Finer market segmentation need not always reduce selection. See, for example, Levin (2001) for a theoretical discussion and Brown, Duggan, Kuziemko, and Woolston (2014) for an empirical application to Medicare.

2 “Feasible” here means subject to the constraint that costs are unobservable or cannot be used in pricing. Below, constrained efficiency means relative to the stated pricing restriction.

3 Even assuming identical insurable losses, identical risk preferences, and identical CARA utility, consumers with different wealth would display differential demand for insurance if bankruptcy protections limited the downside exposure for consumers with little wealth (Mahoney (2015)). In addition, Ericson and Starc (2015) provide some direct evidence of heterogenous price sensitivity in insurance markets across identifiable groups of consumers.

4 Einav and Finkelstein (2011) note the importance of demand heterogeneity in insurance markets but do not consider the possibility of preference-based pricing. Bundorf, Levin, and Mahoney (2012), one of the closest prior papers, shows that when willingness-to-pay varies among consumers carrying the same objective risk, a first-best allocation is infeasible as long as asymmetric information on costs exists. That paper, similarly, does not explore prices linked to consumer preferences. Only one prior paper, Glazer and McGuire (2011), considers a policy that conditions insurance prices on some measure of a consumer’s willingness-to-pay. That paper presents a stylized model of income-based premiums in an exchange setting but offers no empirical application.

5 The perspective of this paper is one of static efficiency, which follows most of the prior literature and permits a focus on the heterogeneity phenomenon of interest. Dynamic efficiency under uniform pricing is more complex to assess due to reclassification risk (Handel, Hendel, and Whinston (2015)). The dynamic efficiency issue might be of secondary importance in this setting in any case: Age-based price discrimination is the primary empirical focus below, and as Handel, Hendel, and Whinston (2015) note, there is no reclassification risk associated with the deterministic process of aging.

6 Compared to Einav, Finkelstein, and Levin (2010), there are several differences here: The risk distribution here (G(s∣δ)) is continuous rather than discrete, consumer characteristics here are partitioned into two vectors (δ and ψ) rather than one to highlight that preferences and constraints enter valuations but not costs, and the setup here ignores moral hazard so as to focus on the selection issues that are the primary interest of the paper. I nonetheless discuss the implications of moral hazard for the empirical exercise. See Section 4.2.

7 For example, consider the choice between two plan options j and k. For plan j to be the efficient choice for individual i, it must be that $v\left(p_{j},\phi_{j},\delta_{i},\psi_{i}\right)-c\left(\phi_{j},\delta_{i}\right)\geq v\left(p_{k},\phi_{k},\delta_{i},\psi_{i}\right)-c\left(\phi_{k},\delta_{i}\right)$.

8 For expositional simplicity, I choose joint distributions of (v,c) that yield linear demand curves, but the intuition applies more generally. Also for simplicity, I discuss the figure as representing a choice between purchasing a single available contract and no purchase, but the figure is consistent with a multicontract setting after a simple transformation. To transform the multicontract case into the same terms, define k∗ as the plan generating the second highest surplus within the choice set. Then rearrange the efficiency condition $v\left(p_{j},\phi_{j},\delta ,\psi \right)-c\left(\phi_{j},\delta \right)\geq v\left(p_{k*},\phi_{k*},\delta ,\psi \right)-c\left(\phi_{k*},\delta \right)$ as $\left(v\left(p_{j},\phi_{j},\delta ,\psi \right)-v\left(p_{k_{*}},\phi_{k*},\delta ,\psi \right)\right)\geq \left(c\left(\phi_{j},\delta \right)-c\left(\phi_{k*},\delta \right)\right)$, and finally redefine $\tilde{v}$ and $\tilde{c}$ as these differences to get the scatter points $\left({\tilde{v}}_{i},{\tilde{c}}_{i}\right)$ and the efficient take-up condition ${\tilde{v}}_{i}\geq {\tilde{c}}_{i}$. Finally, in these plots I make the standard assumption that price is separable from other aspects of insurance valuation; that is, I write $v_{i}$ to represent $v\left(\phi_{j},\delta_{i},\psi_{i}\right)$, rather than $v\left(p_{j},\phi_{j},\delta_{i},\psi_{i}\right)$. A more general formulation would require three-dimensional plots to accommodate changes in $v_{i}$ with prices. The plots in Figure 1 and can be viewed as two-dimensional slices from such plots for some fixed price.

9 Alternatively, the figures can be interpreted as plotting a measure of consumers, with scatter points within each panel representing the preferences and expected costs of point masses of consumers.

10 In other words, although the industry marginal cost curve may have an arbitrary shape, there is no association between the cost of insuring a particular consumer and his/her valuation of the contract.

11 In this paper, the focus is on the social planner’s problem of optimal pricing so as to highlight the phenomenon of interest. Nonetheless, the competitive equilibrium without regulator intervention can be seen in Figure 1 at the intersection of the implied average cost curve and demand.

12 Although v and c are positively correlated overall in Figure 2, the relationship is not monotonic, and there are points i and $i^{\prime }$ for which $v_{i}>v_{i^{\prime }}$ even though $c_{i}\leq c_{i^{\prime }}$.

13 The potential importance of nonmonotone cost curves is acknowledged in the recent literature (see, for example, Einav, Finkelstein, and Cullen (2010) and Einav and Finkelstein (2011)), though the phenomenon is often set aside for tractability in modeling.

14 Here, “preference” is merely shorthand for demand heterogeneity that is conditional on costs. The model captures a broad notion of heterogeneity that includes differences in preferences, differences in constraints, and differences in higher moments of the risk distribution, any of which could affect demand conditional on expected insurer costs.

15 Throughout the paper I discuss the role of demand heterogeneity conditional on cost as determining the social optima, though with adequate support in the valuation–cost space, heterogeneity in demand conditional on costs implies heterogeneity in cost conditional on demand and vice versa. With that in mind, the phenomenon could be recast as one of cost heterogeneity conditional on demand. See, for example, Figure 4, where valuation differs conditional on marginal cost, and marginal cost differs conditional on valuation.

16 Differences in enrollment imply differences in valuations (though the converse is not necessarily true): If valuations were identical, and because prices are identical by construction, choices would have to be identical. Therefore, $\rho_{I(j),z|c}\neq 0$ implies $\rho_{v,z|c}\neq 0$.

17 There is a knife-edge possibility that uniform prices could be optimal even if (4) is satisfied. See the discussion in Appendix A.1.

18 See Van de Ven and Ellis (2000) and Breyer, Bundorf, and Pauly (2011) for overviews.

19 The compensation $\left({\text{RA}}_{i}\right)$ is typically equal to the difference between expected costs as predicted by the regulator (${\hat{c}}_{i}$) and mean costs in the population $(\bar{c}):{\text{RA}}_{i}={\hat{c}}_{i}-\bar{c}$. Perfectly functioning risk adjustment can flatten the firm’s perceived cost curve. Optimal uniform pricing requires an additional refinement: a lump-sum subsidy that serves to set the level of the firm’s perceived constant average cost at the level where demand and unadjusted marginal cost would otherwise intercept. Risk-adjusted payments to achieve the constrained optimum under uniform pricing would take the form ${\text{RA}}_{i}^{*}={\hat{c}}_{i}-\bar{c}+\eta$, where η is this appropriate lump-sum subsidy, which could be financed through a flat tax across all plans.

20 The source is the Medstat Commercial Claims Database, accessed via the National Bureau of Economic Research (NBER).

21 The coinsurance rate is the marginal price faced by the consumer once the deductible has been met. Both the deductible and the coinsurance are counted toward the out-of-pocket maximum. The cost sharing rules in Table 1 apply only to in-network services. Out-of-network services, which comprise a small minority of all claims, involve higher cost sharing in both plans.

22 When prices are needed in the structural choice model below, they are constructed by applying typical employee contribution formulas to average plan costs.

23 Singly enrolled employees are the simplest group in which to draw a clean comparison of demand differences conditional on age and sex. Enrolled families, in contrast, are mixed demographic groups but must make a common health plan choice. Roughly one third of enrolled employees enrolled only themselves rather than themselves plus one or more family member. These employees may nonetheless have spouses and children who receive insurance through other channels, such as the spouse’s employer.

24 Some fraction of the spending differences across plans is likely due to moral hazard. Given the common range of estimates for the elasticity of healthcare consumption with respect to out-of-pocket price, this would be a small fraction. For example, the RAND Health Information Exchange (HIE) (Manning, Newhouse, Duan, Keeler, and Leibowitz (1987)) estimates this elasticity to be −0.2. Applying this parameter and the 10% coinsurance difference between L and H to the average health spending in L would imply that the counterfactual spending of plan L enrollees if they were observed instead in plan H would be $3028(=$2672+$2672×(−0.10/0.15)×(−0.2)). Moral hazard would thus explain little of the observed spending difference between plans H ($6090) and L ($2672).

25 See Angrist and Pischke (2008, Chapter 3). If the conditional expectation functions are linear, then the OLS regression $I_{j_{i}}=\sum_{z\in Z}B_{z}I_{z_{i}}+\beta_{c}c_{i}+\epsilon_{i}$ is just the conditional expectation. Otherwise, the OLS regression gives the best linear predictor of $I_{j}$ given (c,z).

26 In other words, the point of view in the regression is that of a social planner or risk-neutral insurer who is concerned with plan choice as a function of cost to insure, but not necessarily with the preferences or budget constraints that generate demand. Einav, Finkelstein, and Cullen (2010) summarize this point about sufficient statistics in welfare analysis in the following way: “Different underlying primitives (i.e., preferences and private information...) have the same welfare implications if they generate the same demand and cost curves.” The only set of explanations for which this fails to hold is if consumers have behavioral biases or incorrect subjective risk assessments. In that case, there is no clear correspondence between revealed preference and social welfare, and in general there is no widely accepted solution to welfare analyses in such cases.

27 Inputs into the ACG from my data include age, sex, pharmacy spending, medical (nonpharmacy) spending, inpatient stays, outpatient visits, emergency room (ER) visits, all International Classification of Disease (ICD-10) diagnosis codes, all Current Procedural Terminology (CPT) codes, national drug codes of all prescriptions filled, and places of service.

28 Alternative expected cost measures, including the predicted values from my own regressions of current expenditure on past expenditure, run separately by age and sex, also produce similar results to those shown in the next section. Unsurprisingly, less of the variance in realized healthcare costs is explained by these alternative predictors.

29 For example, the Johns Hopkins ACG tool could overpredict expected spending for the young and underpredict it for the old. If so, it would have the effect in Figure 6 of (spuriously) spreading the regression lines away from each other. A more subtle problem would occur if the ACG prediction had no differential bias across groups but suffered from measurement error. In that case, estimates of the age dummies would be biased as well, in the sense of no longer representing age effects that are independent of expected costs. For example, in the extreme, if the health risk variable were purely noise, one would expect large age coefficients, even if the true age effects were zero, because the age coefficients would simply capture the correlation between age and average risk. The test in Table 4 rules out that this alternative hypothesis was generating the results.

30 The regression is $C_{it}=\alpha +\eta {\hat{C}}_{it}+\sum \beta_{a}I_{a}+\mu_{it}$, which should yield a slope η of 1 and an unconstrained intercept α equal to 0. Most importantly, controls for age group indicators $I_{a}$ should yield small and in-significant coefficient estimates.

31 It is unsurprising that age has no residual predictive power in this regression, given that age is already included as an input to the ACG prediction of ${\hat{C}}_{it}$.

32 The p-values on the difference between the estimated slope and 1.00 are never smaller than 0.20.

33 See footnote 24 for an illustrative calculation that considers counterfactual plan H spending of plan L enrollees, accounting for moral hazard.

34 Plan elections are made in fall of the year prior to the plan year.

35 It is not expected that the coefficients on age indicators will be invariant across the columns of Table 5. Only conditioning on expected costs, as in column 1, removes the mechanical correlation between age and the cost variable that theory predicts should drive plan choice. The coefficients on age indicators in columns 2–5 will reflect both the partial correlation between age and choice, holding expected costs fixed, and the part of variation in age that is correlated with the omitted expected cost variable.

36 It is unknown whether a default option existed for the first year of health plan enrollment. It would be typical to default enrollees into the lower cost plan, L.

37 Because employers typically contribute significantly to premiums, the relevant decision for most employees is which plan to purchase rather than the choice of gaining insurance coverage versus not. In the model, this is represented by the outside option being defined as choosing the lower coverage plan, L. The choice of remaining uninsured is not modeled, and employees choosing to remain uninsured are not observable in the data.

38 CARA preferences are typically an explicit or implicit assumption in the literature. Assuming CARA allows direct comparison of my estimate of risk aversion with other studies. See Table 7.

39 Wealth, which is not observed empirically, plays no role in plan choice under CARA preferences. This is apparent in the inequality that determines the take-up of plan $H:V_{iH}>V_{iL}$, which is $\int -\frac{1}{\gamma }e^{-\gamma W_{i}}$. $e^{\gamma \left(P_{H}+\text{OOP}-\delta_{o}H-\sum_{a}\delta_{a}I_{a}\times H-\delta_{F}I_{F}\times H-\epsilon_{iH}\right)}dF_{iH}(\text{OOP})>\int -\frac{1}{\gamma }e^{-\gamma W_{i}}\cdot e^{\gamma \left(P_{L}+\text{OOP}\right)}dF_{iL}(\text{OOP})$. Here the wealth terms cancel. For the same reason, only the price *difference* between plans L and H is needed for estimation.

40 Log normal with a point mass at zero is a commonly used parameterization of medical risk. A mixture of a point mass with a two parameter Weibull—an alternative that is less widely used—fits these data less well. It underestimates the right tail of the distribution.

41 To focus on the welfare implications of the demand heterogeneity of interest here, a maintained assumption is that healthcare consumption will be identical under either plan. This is equivalent to an assumption of no moral hazard. See footnote 24 for a back-of-the envelope calculation regarding the probable importance of moral hazard relative to selection in this setting.

42 Simulation is needed to calculate likelihood values because there is no closed-form expression for the choice probabilities implied by Equation (7).

43 The method here generates different effective “prices” by exploiting differences in out-of-pocket spending distributions across individuals. Unlike the gross demand differences across groups that are identified in Section 4, premium price responsiveness in the discrete choice model, which depends on estimating risk aversion, can only be identified if spending distributions differ across individuals, at least in higher moments. In contrast, with quasi-experimental price variation as in Einav, Finkelstein, and Cullen (2010), it would be possible to identify demand differences across groups without relying on variation in the OOP distributions.

44 For example, the expected utility assumption would be violated if, as a behavioral/psychological phenomenon, consumers viewed spending $50 on a monthly premium differently from spending $50 on a physician bill.

45 I also tried an alternative specification that allowed the risk aversion coefficient to vary by age, rather than including interactions of age and plan H dummies in the money term. In practice, the model in which age entered via risk aversion did not fit the data well. This is likely because preference for H within each age group was not correlated with dispersion in the OOP spending distribution, which such a specification would require. A separate benefit of the formulation in Equation (7) is that the estimates yield easily interpretable differences in valuation that are measured in dollars.

46 For example, in 2007 for employee-only coverage in large firms, workers on average contributed 16% of the premium, with firms contributing 84% (Kaiser Family Foundation and the Health Research and Educational Trust (2007)).

47 Starting from the average total spending in L and H from Table 2, the employer’s share of the average cost difference is $[6900−300−(6900−300)0.1]−$[2672−500−(2672−500)0.2]=$3473;20% of this is $695.

48 The clearest way to see this is to note that in the term $e^{\gamma \left(P_{H}+\text{OOP}-\delta_{o}H-\cdots \right)}$, $\delta_{o}H$ and $P_{H}$ enter identically. Because men and women and younger and older consumers face the same (unobserved) price, the $\delta_{a}$ and $\delta_{F}$ coefficients are not affected when $\delta_{o}$ moves one-for-one with the assumed $P_{H}$.

49 The plan H intercept indicates that, overall, agents make choices as if contract H were priced at $832 more than the assumed price. There are several ways to interpret this coefficient. First, and most obvious, actual employee contributions to premiums are not observed, so the assumed price difference between contracts L and H may be wrong, and the coefficient may partially reflect this. Alternatively, the CARA model may fail to adequately describe all the complexities of health plan choice, in which case the plan H coefficient is merely a convenient modification that allows the model to better fit choice patterns in the data. In terms of fit, a likelihood ratio test strongly rejects the null model in which the plan H intercept is not included (p<0.0001).

50 Note that in the welfare framework here it will be efficient for people with sufficiently large idiosyncratic tastes for plan L to enroll in the lower coverage option even when it is less expensive than the higher coverage option. These taste shocks merely rationalize the patterns in which many low-cost types are observed to choose the higher coverage option and many high-cost types are observed to choose the lower coverage option. This feature of the exercise highlights an important limitation of relying on revealed preference and neoclassical welfare analysis in this context.

51 Specifically, ${\text{CE}}_{ij}$ is found by equating the utility of the sure amount to the expected utility of the lottery, and solving for the sure amount: $-\frac{1}{\gamma }e^{-\gamma \cdot {\text{CE}}_{ij}}=\int -\frac{1}{\gamma }e^{\gamma \cdot \left(P_{j}+\text{OOP}-\delta_{o}H-\sum_{a}\delta_{a}I_{a}\times H-\delta_{F}I_{F}\times H+\epsilon_{iH}\right)}dF_{ij}(\text{OOP})$.

52 In panel B, I integrate over the error distribution to generate expected demand for each individual at each price.

53 To put the dollar amounts represented along the horizontal axis in context, recall that the relevant marginal cost from a social welfare perspective is the difference in the insurer’s expected cost of enrolling the consumer in plan H instead of plan L. For a consumer with the average level of healthcare charges, $3865, this marginal cost (MC) would be approximately $\text{\$}220:\text{MC}(\text{\$}3865)=c_{H}(\text{\$}3865)-c_{L}(\text{\$}3865)=\text{\$}[3865-300-0.9\times (3865-300)]-\text{\$}[3865-500-0.8\times (3865-500)]=\text{\$}220$. The simple calculation in this note ignores the concavity of cost-sharing rules, applying the cost-sharing rules to the expected level of healthcare utilization rather than taking the expectation over the distribution of insurer costs. The actual calculation of the marginal cost curves in Figure 7 fully accounts for the nonlinearity of cost-sharing rules and integrates plan costs over the individual-specific health risk distribution.

54 For comparison, Appendix Figure A1 draws the type of demand homogeneity across consumer types that is almost always implicitly assumed in the literature. In that figure, a single price sorts efficiently since consumer types (men and women, young and old, rich and poor) differ in demand on average only because they differ in insurable risk on average.

55 In markets with heterogeneity, marginal costs are better described as clouds than curves, so rather than attempting to graphically identify a single intersection point within that cloud, optimality is determined by numerically searching over prices and identifying the price that maximizes social surplus.

56 There is no important conceptual difference between an employer or social planner who sets the efficient incremental cost for enrolling in H and the regulator who implements the same efficient price differential in a competitive market setting via risk adjustment and/or subsidizing premiums. In the constrained optimal uniform pricing scenario, the direct-to-consumer subsidies are assumed to be uniform. Otherwise, the subsidies would be effectively generating different prices across the consumer groups, in violation of the uniform price constraint.

57 The demand estimation above is not appropriate for the task of simulating this loss directly. While the risk aversion parameter is a primitive of the utility function that could be applied to the choice of plan H versus no insurance, the δ and ∈ parameters are preferences that are specific to the choice between plan L and plan H.

58 The White House has made statements in the same spirit: “We passed the Affordable Care Act, which . . . says women can’t be charged more for health insurance just because they’re women” (Barack Obama, March 16, 2016). The European Union’s Justice Commissioner has made similar remarks: “Gender equality is a fundamental right in the European Union and the Court of Justice made clear that this also applies to insurance pricing” (European Commission, December 20, 2012).
