# Supplementary material for: Demand heterogeneity in insurance markets: Implications for equity and efficiency
Source: Quant Econom. Author manuscript; Available in PMC 2024 Jul 12. (PMC11244824; doi:10.3982/qe794)
Supplement: Supp Material [file NIHMS1908621-supplement-Supp_Material.pdf]

Supplement to "Demand heterogeneity in insurance markets: Implications for equity and efficiency"

(*Quantitative Economics*, Vol. 8, No. 3, November 2017, 729–975)

Michael Geruso
Department of Economics, University of Texas at Austin and NBER

A.1. Proof of welfare-improving price discrimination

Consider an insurance market in which consumers are associated with a type z that represents a signal of insurance valuation, conditional on marginal cost. Assume that within potential submarkets defined by types z, both the demand and marginal cost curves are strictly decreasing (monotonicity), that demand is more steeply sloped than marginal costs, and that demand and marginal costs cross exactly once (single crossing). Let the rank order of consumer valuations across types z hold conditional on any level of costs. Heterogeneity of this form implies there is correlation between v and z conditional on c.

Under these conditions, if the efficient take-up of insurance is strictly between 0 and 1 within each of the submarkets and if there exists some common support across the z submarkets in the continuous distributions of marginal cost, then optimal prices differ across types z. That is, there is welfare-improving price discrimination along the characteristic z, relative to the best uniform price.

To prove welfare-improving price discrimination, define $v_a(c)$ and $v_b(c)$ as willingness-to-pay conditional on costs for a fixed contract among consumers in groups a and b, respectively. By the assumptions above, these functions are one-to-one mappings of costs to valuations. Define the group $z = a$ as the group for which willingness-to-pay conditional on costs is higher and define $z = b$ as the remainder of the market, so that $v_a(c) > v_b(c)\ \forall c$.

Except for the degenerate cases where $v_a(c) > v_b(c) > c\ \forall c$ (all enrollees in the market efficiently insured) or where $c > v_a(c) > v_b(c)\ \forall c$ (all enrollees in the market efficiently uninsured), there exists some level of costs, $\underline{c}$, for which $v_a(\underline{c}) > \underline{c} > v_b(\underline{c})$. That is, there exists some level of costs at which it is efficient to insure the a types but not the b types. A simple version of this case that assumes linear $v(c)$ functions is displayed in Figure 2.

Michael Geruso: mike.geruso@gmail.com

Copyright © 2017 The Author. Quantitative Economics. The Econometric Society. Licensed under the Creative Commons Attribution-NonCommercial License 4.0. Available at http://www.qeconomics.org.
DOI: 10.3982/QE794

For group a consumers with costs $\underline{c}$, only prices $p \leq \underline{c}$ sort efficiently. For group b consumers with costs $\underline{c}$, only prices $p > \underline{c}$ sort efficiently. Therefore, no uniform price can sort all consumers in both groups efficiently.

Now consider setting separate prices for groups a and b. By the single-crossing and montonicity assumptions, within groups a or b all consumers can be sorted efficiently with the correct group-specific price. This within-group case is a standard case considered in the literature (see, e.g., Einav and Finkelstein (2011)). Therefore, optimal group-specific prices that sort all consumers efficiently are welfare-improving relative to *any* uniform price because no uniform price can sort consumers with costs $\underline{c}$ efficiently.

Note that the case above is constructed so that the types z break up the market into submarkets with monotone marginal cost curves. In this case, the types with higher valuations optimally face lower prices. While the simplicity of this case is instructive, a more conceptually difficult case occurs when one allows for z to be only an imperfect signal of demand conditional on costs, so that submarkets defined by z do not contain strictly decreasing, monotone marginal cost curves. In such cases, optimal pricing could be higher, lower, or—in a knife-edge case—equal for z types with higher valuations, depending on the exact pattern of the residual demand heterogeneity. See Figure 4 for an example of the complex case in which optimal prices are higher for the higher-valuation types.

A.2. Worker and firm characteristics

The firm is a manufacturer of consumer nondurables, with plant locations throughout the United States. Files are purged of information that could be used to identify the employer or employees, though the "anonymized" employees are traceable across plan years. Workers are predominately male. Most are hourly. Workers who decline coverage are not in the data. Although no information is known regarding employees who waive coverage, data from the Kaiser Family Foundation (2007) Survey of Employer Health Benefits indicates that 15% of workers in firms of similar size and in the same industry (consumer nondurables) around the study period waived coverage.

A.3. SMLE estimation details for Section 6

Simulated maximum likelihood estimation (SMLE) is used to estimate Equation (7). Given the choice model outlined above, there is no closed-form expression for the choice probabilities. Therefore, simulation is needed to calculate likelihood values. Here, I assume a normal error that enters the model nonlinearly, but even if the error structure admitted a closed-form solution to the choice probabilities (as would be the case, for example, with an additive extreme value error, which yields logit probabilities), simulation would still be necessary so as to numerically integrate over the risk distribution $F_{ij}(\text{OOP})$.

Estimation begins by fixing the parameter vector θ and taking Q draws from the distribution of out-of-pocket expenses, $F_{ij}(\text{OOP})$, to calculate an expectation over health risk. Conditional on a set of values from the parameter vector (including a single draw of the error term ϵ_{ij}), the expected utility for person i in plan j is

$$E\left[V_{ij}^r\right] \approx \frac{1}{Q}\sum_{q=1}^{Q} -e^{\gamma\cdot(P_j+\text{OOP}^q-\delta_o H-\sum_a \delta_a I_a\times H-\delta_F I_F\times H-\epsilon_{ij}^r)}. \tag{9}$$

The superscript r denotes a draw from the normal error term.

A basic accept–reject (AR) simulator would assign a value of 0 or 1 as the choice probability of plan j depending on whether j generated the highest utility given the simulated value of the error, ϵ_{ij}^r. Here, I use a smoothed AR simulator. For each draw r, a smoothing function is applied to ensure that the probability falls strictly between 0 and 1, avoiding problems otherwise arising from flat portions of the log-likelihood function. I employ a logit smoothing kernel that follows Train (2009):

$$S_{ij*}^r \equiv \Pr\left(j=j^*|\theta^r, n\right) = \frac{\exp\left(E\left[V_{ij*}^r\right]/\lambda\right)}{\sum_J \exp\left(E\left[V_{ij}^r\right]/\lambda\right)}. \tag{10}$$

This smoother approaches an unsmoothed AR simulator as λ approaches 0.[1] The output of the smoother is the probability of selecting a plan, conditional on values from the parameter vector, including a single draw from the normal error term. So as to calculate the simulated choice probability, many draws are taken from the error (still for a fixed parameter value of σ_ϵ). Averaging the smoother values across these draws yields the simulated probability of person i choosing plan j:

$$\breve{P}_{ij}(\theta) \equiv \frac{1}{R}\sum_{r=1}^{R} S_{ij}^r(\theta). \tag{11}$$

Finally, the simulated log-likelihood function is defined by

$$\text{SLL}(\theta) = \frac{1}{N}\sum_{i=1}^{N}\sum_{j=1}^{J} d_{ij}\ln \breve{P}_{ij}(\theta), \tag{12}$$

where d_{ij} is an indicator function for the observed choice of each individual. Standard numerical maximization routines are used to search for the maximizing parameter vector. In practice, I set $Q=50$, $R=100$, and $\lambda > 5$.

[1]The appearance of a logit formula at this step has nothing to do with assumptions on the error term: it is purely a convenient way to ensure that choice probabilities fall strictly between 0 and 1, as in Train (2009).

(A) Homogeneity in (v, c) space

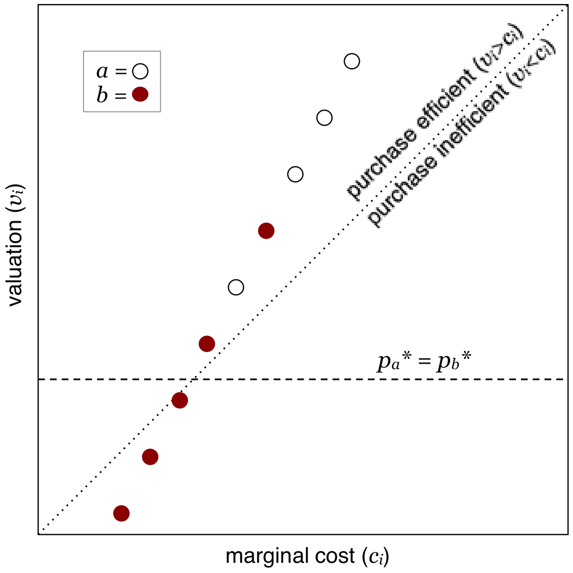

(B) Homogeneity in (P, Q) space

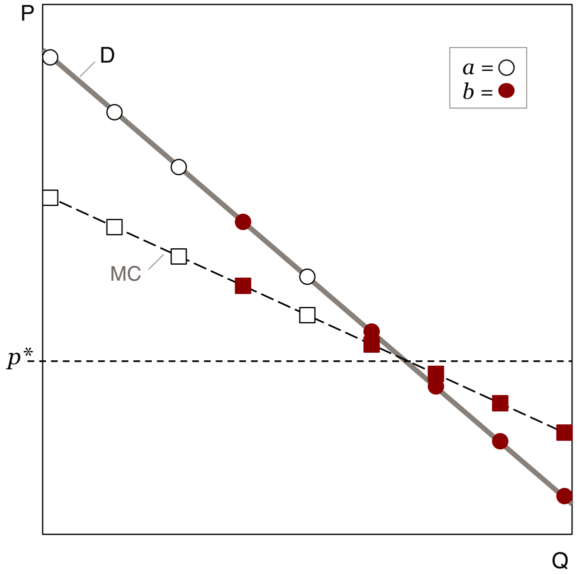

FIGURE A1. Model implied by the common wisdom that uniform prices can sort efficiently. The figure illustrates the model implicit behind the common wisdom that uniform prices can achieve an optimal allocation. Here, a single price sorts efficiently, even though types differ in their costs on average. The common wisdom embodied in the figure implicitly relies on the assumption that consumer types differ only because they differ in the costs they generate, but not demand conditional on costs. See the notes to Figure 1 for additional documentation.

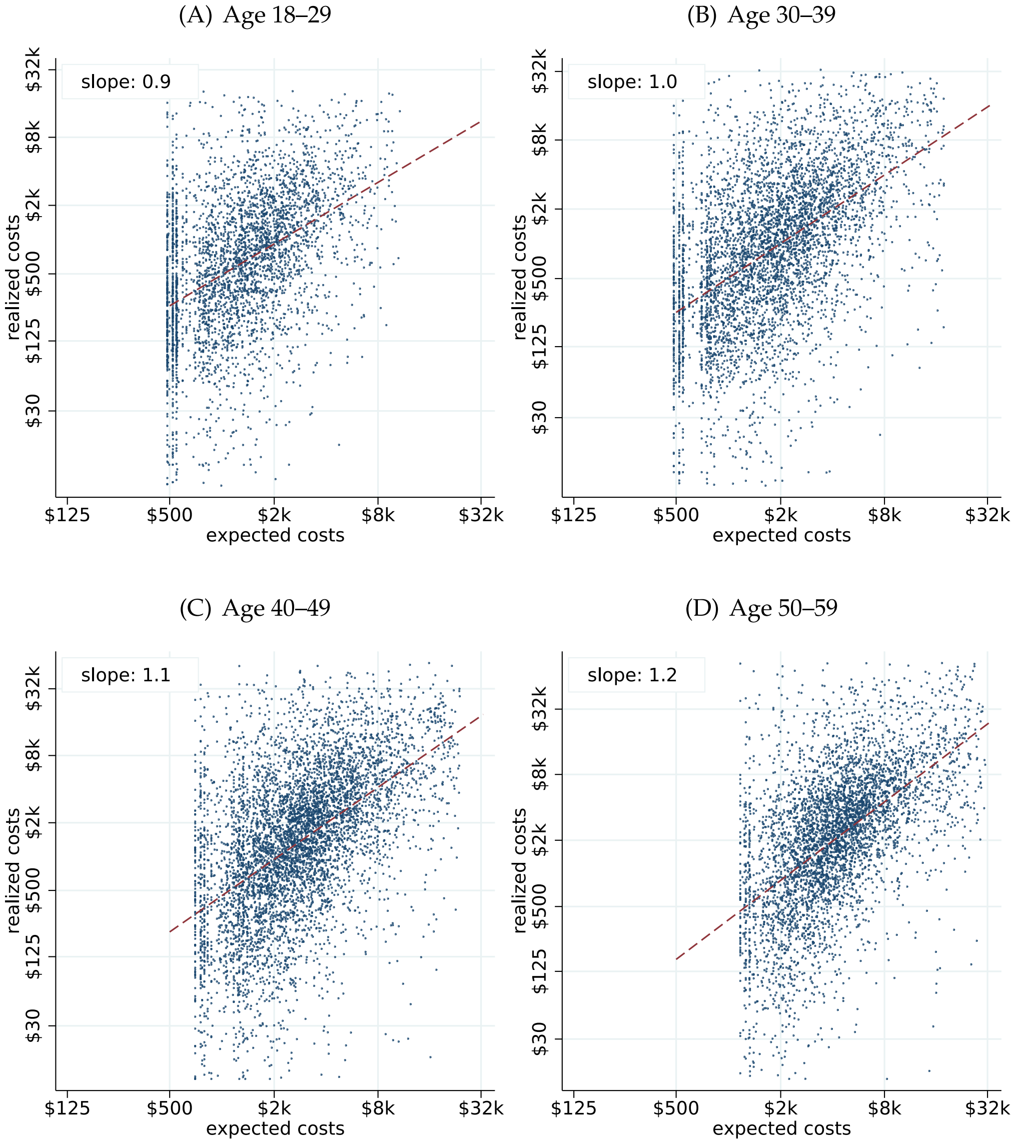

FIGURE A2. Data: Expected costs ($\widehat{C}_i$) and realized costs (C_i). The figure plots scatters of realized healthcare spending versus expected spending, including the insurer and consumer shares. Each panel contains a separate age group. Expected costs are predicted by the Johns Hopkins Adjusted Clinical Grouper (ACG) on the basis of prior year diagnoses and procedures. See the text for full details. The dashed line indicates the intercept and slope from a log-log regression of actual spending on expected spending.

(A) Using ex post realized costs in place of expected costs (perfect foresight approach)

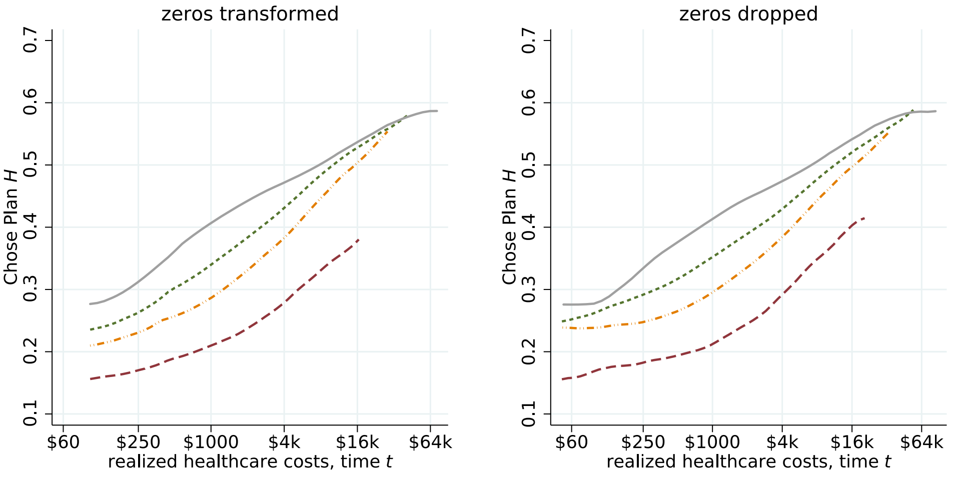

(B) Using last year's costs in place of expected costs (heuristic approach)

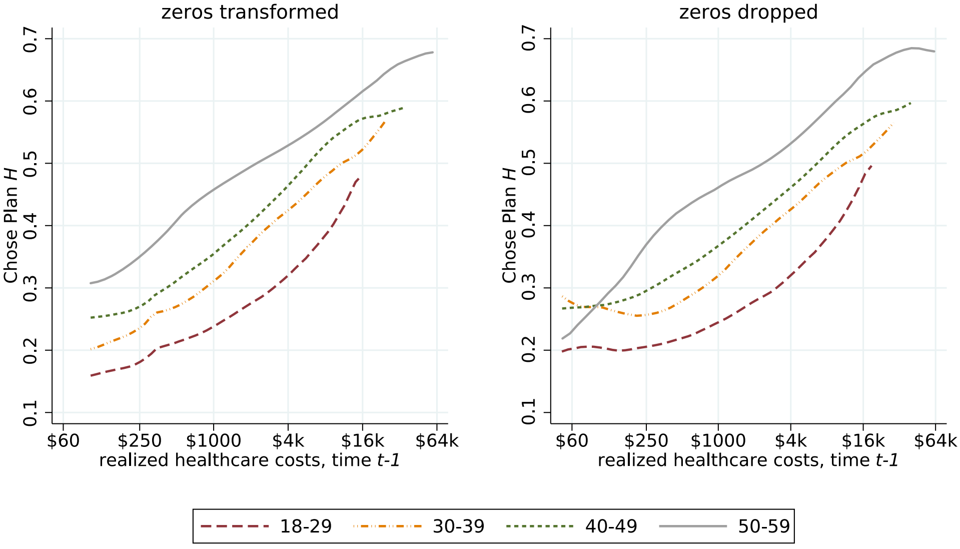

FIGURE A3. Robustness/mechanisms: Revealed demand heterogeneity, alternative cost measures. The figure plots local polynomial regressions of plan choice against expected healthcare consumption. The dependent variable is an indicator for choosing the more generous plan, H. Each panel of the figure exactly replicates panel A of Figure 6, except that along the horizontal axes, alternative cost measures are used. In panel A, the current year's actual realized costs are the conditioning cost variable, rather than an unbiased measure of *expected* costs for the coming year. This would represent perfect foresight on the part of consumers, who choose plans prior to this realization. In panel B, last year's actual costs are used as the conditioning variable. In the left panels, zeros are transformed to allow taking logs. In the right panels, zeroes are dropped from estimation. Consult the text and Figure 6 for additional details.

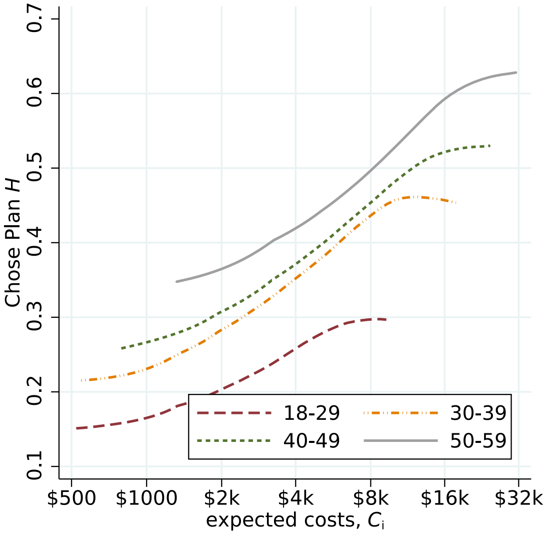

FIGURE A4. Model fit: Simulated plan choices. The figure demonstrates the fit of the expected utility model. The plot replicates panel of A of Figure 6 in plotting local polynomial regressions of plan choice on expected healthcare consumption. In this figure, rather than using the observed plan choice, the dependent variable is the simulated probability of choosing plan H. Simulated choices are predicted by the expected utility model parameters reported in Table 6. See the notes to Figure 6 for additional details.

REFERENCES

Einav, L. and A. Finkelstein (2011), "Selection in insurance markets: Theory and empirics in pictures." *Journal of Economic Perspectives*, 25 (1), 115–138. [2]

Train, K. (2009), *Discrete Choice Methods With Simulation*. Cambridge University Press. [3]

Co-editor Rosa L. Matzkin handled this manuscript.

Manuscript received 2 November, 2016; final version accepted 15 December, 2016; available online 24 January, 2017.
